# A study on the visual behavior and cognitive preferences of female college students in outdoor campus spaces based on eye-tracking

**DOI:** 10.3389/fpsyg.2026.1896634

**Published:** 2026-09-03

**Authors:** Fang Wen, WeiFeng Zhang, Xinzheng Wang

**Affiliations:** School of Architecture and Art, North China University of Technology, Beijing, China

**Keywords:** cognitive preferences, eye tracking, female college students, outdoor spaces on campus, visual behavior

## Abstract

**Background:**

As life pressures rise and opportunities to connect with nature decline, mental health is a major concern for Chinese college students, with campus outdoor spaces supporting their well-being. Female students show heightened sensitivity to visual comfort, safety, and usability, frequently using outdoor areas for rest, socializing, and emotional regulation. Given the close link between these spaces and their campus experiences, female students are a key group for assessing campus space quality from a gender-responsive perspective.

**Methods:**

This study combined eye-tracking technology with subjective evaluations to explore female college students’ visual behavior and spatial cognitive preferences in campus outdoor public spaces. A total of 34 female participants aged 18–23 were recruited, and experiments were carried out at six typical campus outdoor sites under non-direct sunlight conditions. Three types of Areas of Interest (AOIs) and 11 environmental object categories were defined. Wearable eye-trackers synchronously collected visual gaze data, supplemented by questionnaire surveys. With image segmentation and spatial analysis, core eye movement indicators were extracted for quantitative analysis.

**Main findings:**

The results showed that natural landscape elements, especially trees, obtained the highest visual attention in total fixation duration, fixation count and average fixation duration, while functional facilities such as streetlight and stone table attracted less attention. Multi-functional plaza and campus walkway area achieved higher subjective satisfaction, whereas rest and communication areas had lower scores. Correlation analysis verified that attention to natural elements positively correlated with spatial satisfaction (*r* = 0.65, *p* < 0.001), while excessive gazing at road surface and sign negatively correlated with safety and comfort perceptions (*r* = −0.57, *p* < 0.001). Participants urgently demanded better shading facilities, supporting amenities and clear wayfinding.

**Implications:**

This study identifies key environmental factors affecting female users’ visual experience and perceived safety, providing empirical evidence for targeted refined design of campus outdoor public spaces for female users.

## 1 Introduction

University campuses are increasingly becoming comprehensive public spaces that integrate learning, socializing, and recreational activities. The outdoor environment on campus not only serves as a space for circulation and the organization of activities, but also directly influences students’ emotional experiences, behavioral patterns, and how they use the space (Hasan et al., 2025). Research shows that a well-designed outdoor campus environment can effectively alleviate psychological stress, enhance restorative experiences, and strengthen students’ sense of belonging and social interaction (Li, 2024; Elly and John, 2025). Consequently, understanding the quality of campus environments from the user’s perspective has become a key focus in environmental psychology and built environment research.

In recent research exploring campus spaces with a focus on female users, female perceptual characteristics have increasingly attracted scholarly attention. Studies indicate that women typically exhibit greater environmental sensitivity in public spaces, with significant differences observed particularly in terms of perceived safety, visual comfort, and spatial accessibility (Min et al., 2022; Sadeghi et al., 2023; Prada-Trigo et al., 2025). On college campuses, female students constitute a significant user group; their spatial experience is influenced not only by spatial form and environmental factors but is also closely linked to their perception of safety, environmental visibility, and spatial legibility. In recent years, numerous studies have examined the impact of the campus environment on college students from the perspective of psychological and emotional experiences. For example, Peng et al. (2024) found through an emotional evaluation model that factors such as green spaces and spatial layout significantly influence college students’ emotional perceptions, with women exhibiting greater emotional sensitivity to environmental changes. On a broader scale, a review of research on campus open spaces further indicates that natural environmental elements—such as green spaces and water features—can enhance college students’ well-being and mental health (Li and Cui, 2025). In addition to emotional experiences, perceptions of safety are also a key factor influencing women’s spatial behavior. Research indicates that female students are more likely to experience fear of crime or safety anxiety in campus environments, particularly at night or in areas with poor visibility (Min et al., 2022; Sadeghi et al., 2023). Environmental design factors, such as lighting conditions, spatial visibility, and surveillance systems, are closely linked to students’ sense of safety (Huang et al., 2022). Research from a gender perspective has further revealed that women are more likely to adopt avoidance or precautionary behaviors when faced with potentially risky environments, thereby influencing the scope and patterns of their spatial activities (Prada-Trigo et al., 2025). In addition, environmental comfort is also a key factor influencing the experience of campus spaces. For example, studies on the outdoor thermal environment on campus have found that female students are more sensitive to changes in thermal comfort and are more likely to experience thermal discomfort (Zhang et al., 2024). At the same time, a large body of research indicates that natural landscape elements—such as trees and green spaces—not only improve environmental comfort but also promote psychological recovery and stress relief (Liu et al., 2018; Ibes and Forestell, 2022; Aghabozorgi et al., 2025; García-Martín et al., 2025; Jiang C. et al., 2025; Jiang G. et al., 2025).

In terms of research methods, traditional studies have largely relied on subjective evaluation methods such as questionnaires, interviews, or behavioral observation. While these methods can reflect users’ overall perceptions, they struggle to reveal the visual attention processes that occur in real-world environments. With advances in eye-tracking technology, researchers are now able to analyze people’s visual attention patterns in their environment more objectively by recording metrics such as fixation points, fixation duration, and visual trajectories (Lu and Pesarakli, 2023). Current research generally agrees that visual attention is a key prerequisite for the formation of spatial preferences. An individual’s gaze location, duration, and saccade trajectories within an environment not only reflect their information-processing mechanisms but also, to some extent, reveal their interests and preferences regarding spatial elements. For example, Evans and Chamberlain (2024) note that eye-tracking data can effectively identify visual hotspots and reveal the mechanisms underlying landscape preferences through spatial distribution patterns. Similarly, Su et al. (2022) found through empirical research that areas with longer fixation durations and higher fixation frequencies typically correspond to higher ratings of spatial appeal, indicating that eye-tracking metrics can serve as an important objective measure of spatial appeal. In recent years, this method has been widely applied in research on urban landscapes and spatial cognition, and has demonstrated that natural landscape elements tend to attract more visual attention and enhance environmental evaluations (Liu et al., 2018; Aghabozorgi et al., 2025). At the spatial level, natural elements (such as greenery) typically have greater visual appeal and are significantly associated with higher environmental preferences (Zheng et al., 2022). At the same time, factors such as spatial openness, structural clarity, and the organization of pathways also influence the allocation of visual attention, which in turn affects spatial evaluation (Ren et al., 2024). In addition, perceptual elements such as color play a significant role in shaping spatial preferences by influencing emotional experiences (Cho and Suh, 2020). From a cognitive perspective, the formation of spatial preferences is a continuous, progressive process involving environmental sensory input, psychological interpretation of information, and the generation of emotional experiences. Eye-tracking metrics can reflect the depth of information processing; for example, longer fixation times typically indicate more in-depth cognitive processing (Meso et al., 2012). Some studies have also pointed out that, under certain conditions, there is a positive correlation between gaze behavior and preference ratings, indicating that eye-tracking data possesses a certain predictive power (Xiao et al., 2026). With the advancement of virtual reality technology, the integration of VR and eye tracking has further enhanced the controllability and realism of research, enabling researchers to systematically analyze the impact of spatial structures and design elements on visual behavior and preferences (Kuliga et al., 2015). However, existing research still has certain limitations. On the one hand, eye movements do not always align with subjective preferences, suggesting that visual attention cannot fully explain the mechanisms underlying preference formation (Miller, 2024). On the other hand, there is a lack of uniform standards across studies in terms of methodology and metrics, and insufficient attention is paid to specific groups. To date, there has been relatively little empirical research on the visual behavior and cognitive preferences of women in outdoor spaces on college campuses (Chaney et al., 2024). Existing studies have largely focused on urban streets or park environments, and few have combined visual behavior data with questionnaire evaluation data (Ma et al., 2023). Therefore, it is necessary to conduct relevant research in a real-world school setting (Mamedova et al., 2026).

Based on this, this study focuses on outdoor spaces on campus, with female college students as the research population. It uses wearable eye-tracking devices to record their visual behavior and combines this data with subjective questionnaire analysis of spatial cognition and preference characteristics to identify key environmental factors influencing visual attention and spatial experience, thus offering empirical evidence for targeted optimization of campus outdoor spaces for female college students.

## 2 Methods

### 2.1 Research design

**Figure 1:**
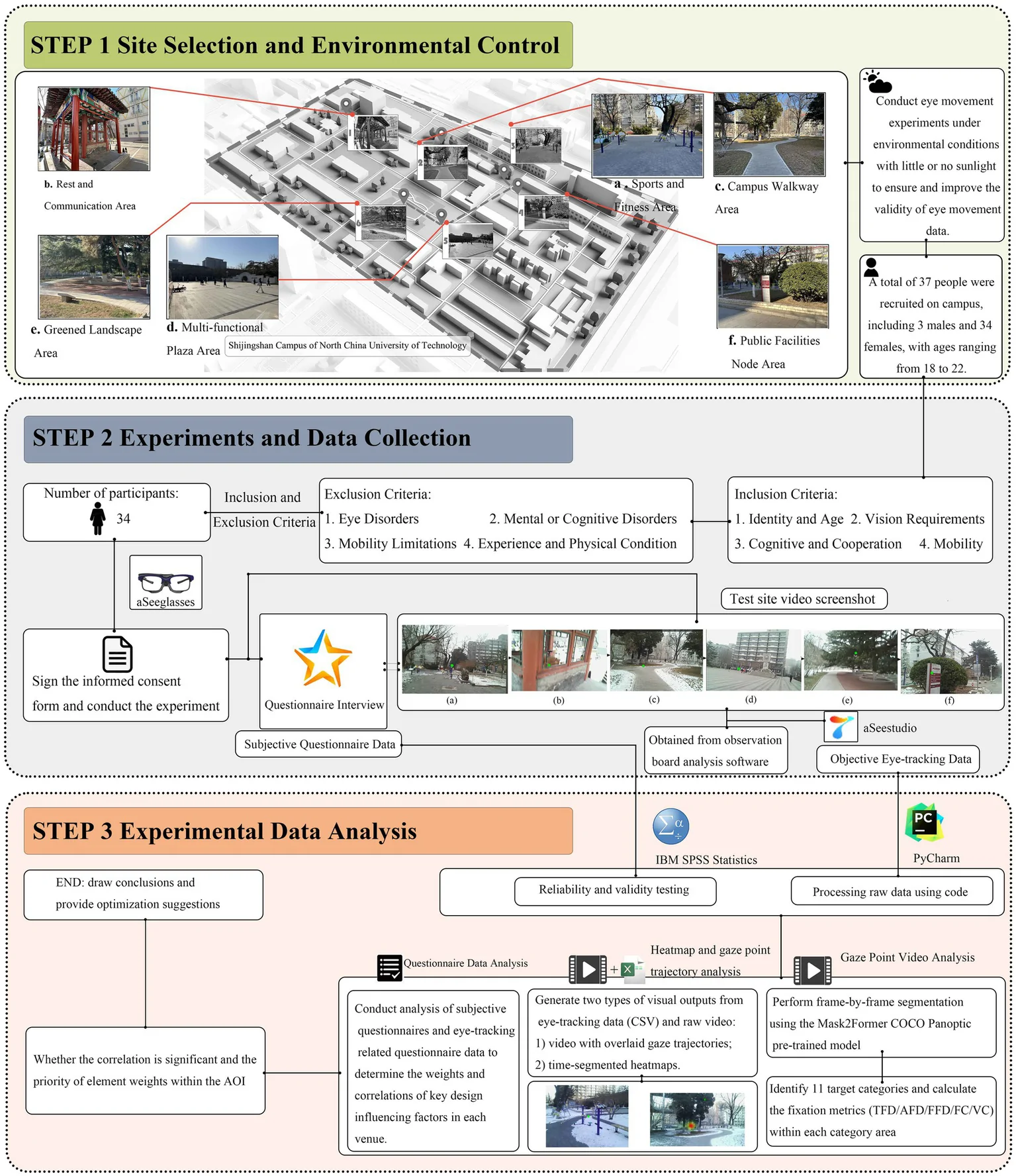
Research framework diagram.

This study employs a mixed-methods approach combining objective behavioral data with subjective evaluations. Using eye-tracking technology as the primary method and supplemented by questionnaire surveys, the study analyzes the visual behavioral characteristics and spatial cognitive preferences of female college students in outdoor campus spaces. The overall research process consists of three phases: selection of experimental sites and environmental control; experimental implementation and data collection; and data processing and statistical analysis (see Figure 1).

### 2.2 Selection of experimental site and environmental control

First, this study selected six typical types of outdoor public spaces on the campus of North China University of Technology as research sites. These were comprehensively screened based on the campus layout, site functional attributes, and the actual activity patterns of female college students to ensure that the research samples align with the study’s theme and that the data are representative and comparable. The outdoor spaces at North China University of Technology are well-equipped and feature a diverse range of landscapes, including recreational green spaces, central multi-purpose plazas, commuting pathways, sports fields, wooded rest areas, and gathering zones near academic buildings. These diverse venues comprehensively address the daily outdoor activity needs of female college students, such as commuting, resting between classes, socializing, and relaxation. The six types of sites selected for this study are all core outdoor areas that female college students visit frequently, are highly likely to linger in, and have a strong preference for in their daily lives. These areas feature a balanced mix of natural and man-made landscape elements, enabling an accurate reflection of their spatial visual perception and usage preferences; however, on-campus venues such as specialized athletic fields, idle logistics areas, construction sites, and remote corners were excluded. These sites have single-purpose functions, low usage rates among women, and numerous disruptive factors; they lack the common characteristics of campus outdoor spaces and are therefore unsuitable for conducting visual-behavioral comparison experiments. To prevent factors such as direct strong light and high contrast between light and shadow from affecting the accuracy of eye-tracking data, this study strictly standardized the lighting conditions of the experimental environment. All on-site tests were scheduled between December 20 and December 30, 2025, and were conducted daily during the low-light periods of 7:00–9:00 a.m. and 4:00–6:00 p.m., when there was no intense sunlight. During these hours in Beijing’s winter, the sun’s angle is low and the light is soft, with no reflections from hard surfaces or strong contrasts between light and shadow, resulting in uniform and stable overall illumination at the site. Experiments were conducted exclusively on clear, windless days, without the use of artificial lighting or temporary shading devices throughout the process. By standardizing the basic lighting conditions, external environmental variables were minimized to the greatest extent possible, ensuring the accuracy and reliability of the eye-tracking data. Second, participants were fitted with wearable eye-tracking devices to experience the campus environment firsthand, while eye-tracking data was collected and subjective evaluation questionnaires were completed; finally, statistical and correlation analyses were conducted on the objective eye-tracking metrics and subjective evaluation data to identify the key environmental factors influencing female college students’ visual attention and spatial perception.

This study uniformly selected winter for all field experiments, as this was the preferred choice after balancing experimental control and theoretical considerations: The Beijing campus is dominated by deciduous trees; in winter, the trees have completely shed their leaves, significantly reducing the pixel proportion of green elements in the image. This effectively mitigates the confounding bias caused by “passive, random gaze fixation resulting from larger areas of greenery in the frame.” At the same time, the clear boundaries of branches, buildings, and roads significantly improve the panoramic segmentation accuracy of Mask2Former; in winter, the sun’s altitude angle is low, and lighting during morning and evening experimental sessions is soft and uniform, free from direct glare, specular reflections, and extreme contrast between light and dark areas. This helps prevent tracking failures and gaze drift in wearable eye-tracking devices, ensuring consistent lighting conditions across the entire site and eliminating the interference of lighting variables on visual behavior; Existing research on landscape-related eye tracking has primarily focused on lush spring and summer scenes, while empirical studies on female students’ visual perception of campus environments during the leafless winter are scarce. The sparse vegetation in winter fully exposes hard infrastructure such as road surfaces and signage, allowing for clear capture of participants’ scanning behaviors driven by safety needs. This enables direct verification of the correlation between excessive fixation on man-made elements and spatial satisfaction and a sense of security, offering unique theoretical insights.

### 2.3 Participant selection

The subjects of this study were female undergraduate students at North China University of Technology. Volunteers were recruited through an open campus recruitment process and screened in strict accordance with predefined inclusion and exclusion criteria. Ultimately, 34 female undergraduate students aged 18–23 were selected as valid participants. The inclusion criteria for participants primarily included being between the ages of 18 and 25, having uncorrected or corrected visual acuity of at least 1.0, and having no visual impairments such as color blindness or color weakness. All participants were in good cognitive condition, capable of accurately understanding the experimental procedures and operational requirements, and able to actively cooperate throughout the on-site testing process. Additionally, participants were required to be in good physical health and possess the mobility necessary for normal outdoor walking and on-site activities. The corresponding exclusion criteria explicitly excluded individuals with severe eye diseases or a history of eye surgery to prevent eye health issues from affecting the quality of eye-tracking data collection; Additionally, students with cognitive or mental impairments, or those with mobility issues preventing them from successfully completing the entire experiment, were excluded. Participants who had previously participated in similar eye-tracking experiments—and who might exhibit a learning effect from prior experimental experience, potentially leading to data bias—were also screened out. Volunteers in poor physical condition or with unstable emotions on the day of the experiment were not permitted to participate. All eligible participants were fully informed of the experiment’s content, procedures, and precautions prior to the formal start of the experiment, and each voluntarily signed an informed consent form, ensuring that the conduct of this experiment complies with academic ethical standards and the scientific requirements of experimental research.

### 2.4 Experimental equipment

The experiment utilized the aSee Glasses wearable eye tracker to collect visual behavior data (Gao et al., 2025) (see Table 1 in the Appendix 1). This device is suitable for dynamic eye tracking in real-world environments, with a sampling frequency of 60–240 Hz and a fixation accuracy of approximately 0.5°. It can record key visual metrics in real time, including fixation points, fixation duration, number of fixations, and gaze trajectories. Data processing and analysis were primarily conducted using aSeeStudio eye-tracking analysis software, the PyCharm programming platform, and IBM SPSS Statistics software.

### 2.5 Definition of regions of interest and experimental procedure

Taking into account the spatial characteristics and landscape elements of the outdoor campus environment, this study identified three categories of Areas of Interest (AOIs): natural landscape areas, architectural and road areas, and recreational and service areas. It further identified 11 types of visual targets to facilitate the analysis of eye-tracking data in relation to environmental elements.

The experiment was organized and conducted entirely by the researchers. First, the purpose and procedure of the experiment were explained to the participants, and their basic information was collected. Subsequently, the participants were fitted with eye trackers and underwent multi-point calibration to ensure the accuracy of data collection. After calibration, the participants followed a predetermined route to experience six types of outdoor campus spaces in sequence, spending approximately 1–2 min at each location. They observed the environment in a natural state, while the eye trackers continuously recorded their visual behavior data. After experiencing all locations, participants completed a structured questionnaire, evaluating each space on a five-point Likert scale across dimensions such as safety, comfort, aesthetics, and convenience. Upon conclusion of the experiment, the eye-tracking data and questionnaire data were exported and consolidated.

### 2.6 Questionnaire design

The “Questionnaire for Eye-Tracking Evaluation of Outdoor Campus Activity Areas” designed in this study aims to systematically assess users’ subjective perceptions of the visual environment (see Appendix 2). The questionnaire comprises 65 items across six dimensions, scored using a 5-point Likert scale, and covers six core areas: fitness equipment, rest and recreation, linear pathways, plazas, green spaces, and public facilities. Results of the reliability and validity analysis show that the Cronbach’s *α* coefficient for the total scale is 0.91, with α coefficients for each dimension ranging from 0.77 to 0.88, indicating that the scale possesses extremely high internal consistency. In the structural validity test, the KMO value was 0.843, and the significance of Bartlett’s sphericity test was less than 0.001. Factor analysis identified six common factors, explaining 62.7% of the total variance, which aligns highly with the original questionnaire structure. In summary, this questionnaire demonstrates excellent reliability and good validity, and exhibits a high degree of alignment with the eye-tracking Areas of Interest (AOI) design. It is capable of effectively and reliably measuring visual behavior and preferences regarding outdoor spaces on campus and is suitable for subsequent data analysis in this study.

### 2.7 Data processing methods

Raw eye-tracking videos and acquired data were processed through a three-stage automated workflow for interpretation, segmentation, and metric extraction. The overall process consists of three core modules: full-frame semantic segmentation, gaze trajectory recognition, and heatmap and eye-tracking metric quantification, supported by a proprietary GUI tool for batch processing.

First, panoramic scene segmentation is performed: the Mask2Former COCO panoptic segmentation pre-trained model is invoked to analyze the experimental videos frame by frame.^1^ The model’s output categories are mapped to the 11 predefined visual regions of interest (AOIs) in this study, generating semi-transparent masks for each AOI that are overlaid on the original footage. This filters out irrelevant categories and precisely delineates the pixel boundaries of each environmental element, providing a scene baseline for gaze attribution determination.

Next, green gaze marker points within the video are identified using HSV color thresholding. Valid gaze center-of-mass coordinates are extracted through morphological denoising, contour filtering, and circularity verification; The program samples the gaze trajectory in 4-s time slices, plots numbered continuous gaze lines on the reference frames of each segment, automatically handles trajectory breaks caused by missing frames, and outputs time-series trajectory screenshot files. Simultaneously, pixel conversion is performed on the standardized gaze coordinates to unify the mapping relationship between microsecond-level timestamps and video frames, and invalid eye-tracking samples with out-of-bounds coordinates or missing markers are filtered out.

Finally, based on the matched AOI regions and valid gaze points, core eye-tracking metrics are calculated in batches: If the center of gravity of the gaze falls within the corresponding region of interest, it is deemed a valid gaze for that region. Consecutive frames within the same region are merged into a single gaze event, and the following metrics are calculated separately: total fixation duration (TFD), average fixation duration (AFD), first fixation duration (FFD), and fixation count (FC) (see Table 2 in the Appendix 1); The tool generates a Gaussian-smoothed heatmap by accumulating gaze density at 4-s intervals (with a color gradient from green to red representing gaze intensity) and simultaneously outputs a processed video overlaid with complete gaze trajectories. All visualization outputs and quantitative metrics are then consolidated and imported into SPSS for subsequent correlation analysis.

The survey data were organized and coded using Excel, and reliability and validity tests were conducted using SPSS software. Subsequently, descriptive statistical analysis was performed on the evaluation results for different types of spaces, and correlation analysis was used to explore the relationship between eye-tracking metrics and subjective evaluations. This approach identified the key environmental factors influencing female college students’ spatial experiences and provided a basis for optimizing outdoor spaces on campus.

### 2.8 Accuracy of the semantic segmentation model and sampling process

In this study, the Mask2Former semantic segmentation model was set up and run in a Python 3.10 environment. The model was pre-trained on the COCO Panoptic dataset, and parameter tuning was performed using a grid search algorithm. Considering that directly transferring a general pre-trained model to a campus setting is prone to recognition biases, we conducted manual validation through stratified sampling specifically tailored to the campus environment to evaluate segmentation accuracy. We selected 420 non-overlapping, non-contiguous frames as stratified samples from six types of campus spaces—academic areas, residential areas, sports areas, green spaces, the street along the campus gate, and support facilities—we selected 420 non-overlapping, non-consecutive frames as stratified samples. Each area category contained 60 frames, which were independently annotated and reviewed by two researchers trained in uniform annotation standards using the same 11-class AOI definitions as the model. Annotation discrepancies were resolved through a two-person consensus process to establish the ground truth. The model’s automatically generated feature masks were compared pixel-by-pixel with the manually annotated consensus data, yielding an average mean IoU (mIoU) of 87.23%, an average Dice coefficient of 92.71, and an overall pixel accuracy of 93.05%. The IoU distribution range for each category was 67.42–97.85%. Since the study required mapping eye-tracking gaze points to corresponding AOI regions, we additionally calculated the matching accuracy at the gaze point level. For gaze points where the automatic segmentation results matched the human annotations for 91.68% (11,590 correct gaze points out of a total of 12,642 valid gaze points) of gaze points matched the same AOI category as the manual annotations. The overall segmentation accuracy supports the objective-area-based eye-tracking analysis in this study.

## 3 Results

### 3.1 Analysis of general characteristics of eye movement behavior and eye movement metrics

#### 3.1.1 Semantic segmentation annotation of raw video

**Figure 2:**
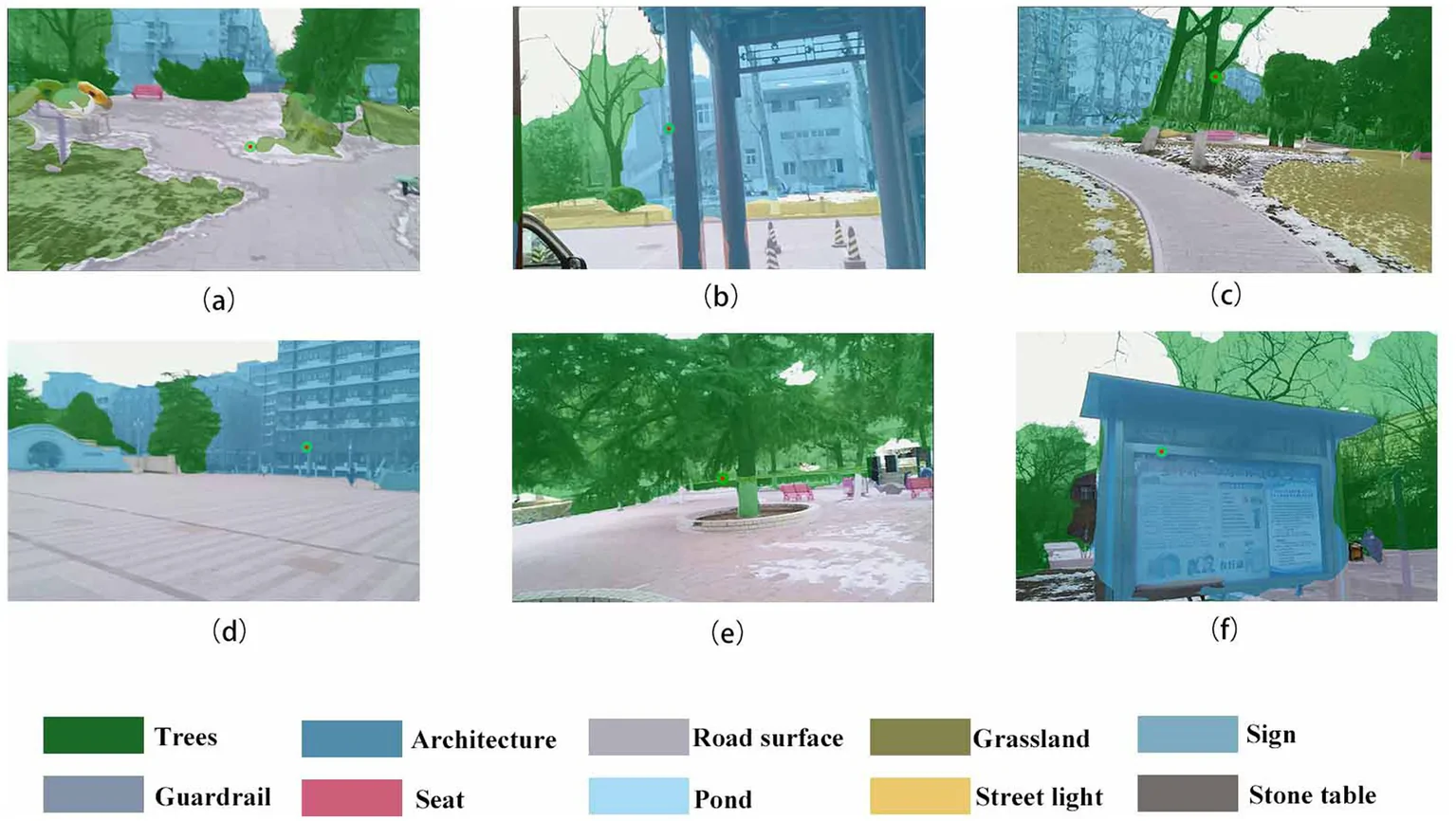
Video semantic segmentation results for six types of sites. **(a)** semantic segmentation annotation of the sports and fitness area; **(b)** semantic segmentation annotation of the rest and communication area; **(c)** semantic segmentation annotation of the campus walkway area; **(d)** semantic segmentation annotation of the multi‑functional plaza area; **(e)** semantic segmentation annotation of the greened landscape area; **(f)** semantic segmentation annotation of the public facility node area.

Figure 2 shows the results of video semantic segmentation for six typical campus locations in this study. Through pixel-level color coding, the spatial distribution of 11 types of environmental visual objects is clearly delineated (Mohareb and Ashraf, 2025). In the legend, dark green represents trees, blue represents architecture, gray represents road surface, olive green represents grassland, and light blue represents sign. The remaining colors correspond to facilities such as guardrail, seat, pond, streetlight, and stone table, thereby providing a general semantic annotation for all environmental elements in the scene (Sun et al., 2022).

Specifically, (a) through (f) correspond to six functional spaces: the sports and fitness area, the rest and communication area, the campus walkway area, the multi-functional plaza area, the greened landscape area, and the public facility node area. It is immediately apparent that the composition of elements varies significantly across different sites: for example, in (a) the sports and fitness area, grassy areas, paved surfaces, and recreational facilities coexist; (d) the multi-functional plaza area is dominated by large areas of hard-surfaced pavement and background architecture; while (e) the greened landscape area centers on tall trees, complemented by elements such as benches and walking paths. It is worth noting that the three types of elements—trees, architecture, and road surface—occupy a significant visual area in all scenarios, which aligns with the results from subsequent eye-tracking data showing that these elements received significantly higher total gaze duration and gaze counts.

In addition, the actual gaze points of the participants are marked with green dots in the figure, further revealing the distribution patterns of visual attention across different semantic regions (Hayes and Henderson, 2021). It can be observed that the participants’ gaze was primarily focused on areas rich in semantic information, such as trees, architecture, and sign, while they paid less attention to elements with low semantic information, such as stone table and streetlight. This scene analysis based on semantic segmentation provides a reliable spatial foundation for subsequent AOI (area of interest) delineation and eye-tracking data analysis, and also offers intuitive visual evidence for the analysis of visual attention differences across different functional environments in this study (Nedaei et al., 2025).

#### 3.1.2 Analysis of eye-tracking metrics for category 3 AOIs

Based on eye-tracking data, this study analyzed the attention allocation patterns for 11 types of visual targets in a campus environment. In accordance with the study design, these targets were categorized into three areas of interest (AOIs): natural scenic area, architectural and road area, and rest and service area. The study systematically examined the differences in visual appeal across these functional spaces (Měkota, 2024).

**Figure 3:**
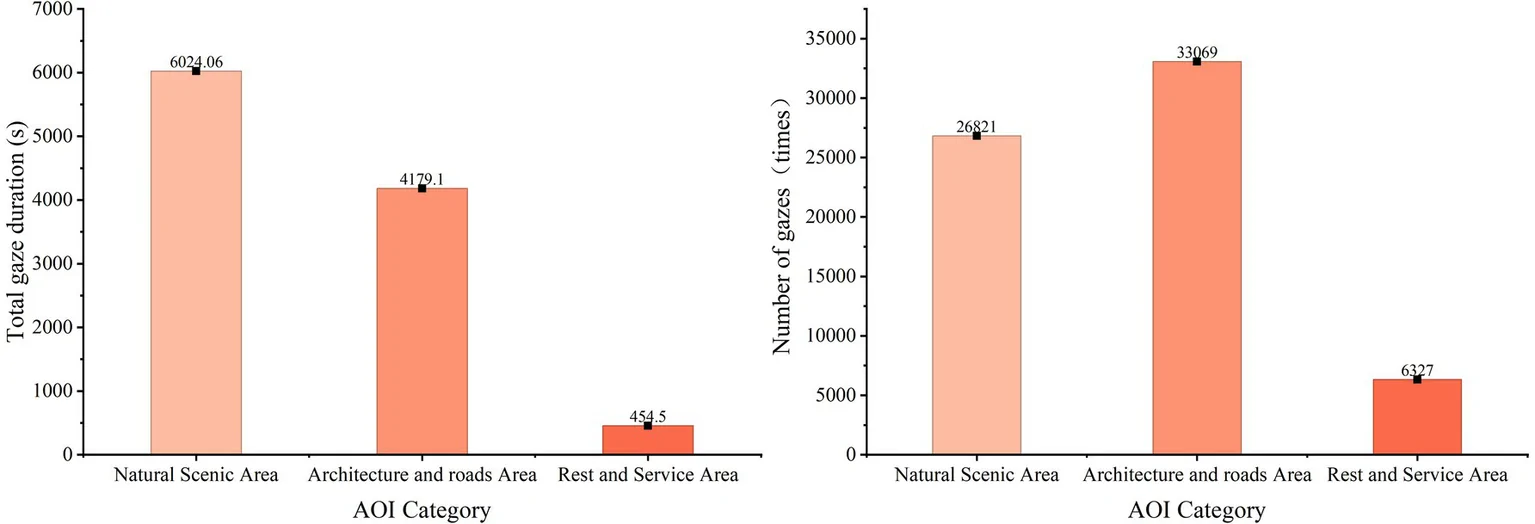
Statistics on total gaze duration and number of gazes for the three types of AOIs.

The results show (see Figure 3) that the visual appeal of natural scenic area (trees, grassland, and pond) was significantly higher than that of other areas, accounting for as much as 56.6% of the total fixation duration. Among these, trees, as the core landscape elements, accounted for 94.5% of the fixation duration in this area, reflecting the strong attraction of natural environments on visual attention (Ji et al., 2026). The architectural and road area (architecture, road surface, and guardrail), as the functional core of the campus environment, accounted for 39.2% of the total fixation duration. Within this area, architecture and pavement received comparable levels of attention, accounting for 49.4 and 46.3% respectively, indicating that the environment’s basic functional elements are also key focal points of visual attention (Huang et al., 2024). In contrast, the total viewing time for rest and service area (streetlight, stone table, sign, seat, and pedestrian) accounted for only 4.3%, indicating a relatively low overall level of attention. Among these, sign and pedestrian received relatively higher levels of attention, while facilities such as seat, streetlight, and stone table had very limited visual appeal (Gerike et al., 2021).

From the perspective of cognitive processing, there were significant differences in the average fixation duration across the three types of AOIs: the average fixation duration in the natural scenic area (0.110 s) and the architecture and road area (0.101 s) was significantly higher than that in the rest and service area (0.029 s), indicating that the visual information in the former two areas requires deeper cognitive processing. The high correlation between the fixation count and total fixation duration (*r* = 0.99) indicates that differences in attention across different AOIs are primarily reflected in fixation count rather than the duration of individual fixations, further revealing the patterns of visual attention allocation in campus environments (Wu et al., 2026).

#### 3.1.3 Analysis of overall characteristics of eye-tracking metrics

Using an eye tracker to collect four core metrics—total fixation duration (TFD), average fixation duration (AFD), first fixation duration (FFD), and fixation count (FC)—we conducted a statistical analysis of visual attention patterns toward 11 types of visual targets (pedestrian, road surfaces, trees, grassland, streetlights, architecture, seat, pond, stone table, guardrail, and sign). The results are shown in the Appendix 1 (Tables 3, 4) and Supplementary material (Table 5):

(1) Overall statistics on eye-tracking metrics for 11 types of visual targets

Trees scored significantly higher than other elements in terms of total fixation duration, average fixation duration, first fixation duration, and fixation count. Streetlight, stone table, and pond had the lowest values for all eye-tracking metrics, indicating that they received virtually no effective visual attention. Road surface and architecture, serving as spatial ground planes and background elements, had moderate levels of fixation count and total fixation duration, reflecting their role in spatial orientation and path guidance (see Table 3 in the Appendix 1).

(2) Analysis of differences in eye-tracking metrics across different visual targets

A one-way ANOVA was conducted on the eye-tracking metrics for the 11 types of visual targets to test for significant differences in these metrics across the various visual targets. The results are shown in the table below (see Table 4 in the Appendix 1). There were highly significant differences among the 11 types of visual targets across all eye-tracking metrics (*p* < 0.001). Among these, trees, as the core representative of natural elements, ranked first in all four metrics: total fixation duration (mean 34.29 s), average fixation duration (mean 0.31 s), first fixation duration (mean 0.23 s), and fixation count (mean 118.47), indicating that they received the highest level of visual attention; Architecture (total fixation duration: 10.63 s) and the road surface (total fixation duration: 8.76 s) ranked second and third, respectively; whereas streetlights (total fixation duration: 0.02 s), stone tables (total fixation duration: 0.01 s), and pond (total fixation duration: 0.15 s) received the lowest visual attention, with all eye-tracking metrics significantly lower than those of other visual targets.

(3) Comparison of eye-tracking metrics across different venue types

A one-way ANOVA revealed significant differences across all eye-tracking metrics among the six site types (*p* < 0.001) (see Table 5 in Supplementary material): the total fixation duration, average fixation duration, and fixation count in the campus walkway area and the greened landscape area were significantly higher than in the other areas. Among these, the campus walkway area had the longest total fixation duration (mean 228.65 s) and the longest average fixation duration (mean 0.38 s); The rest and communication area had the lowest values for all eye-tracking metrics, with a total fixation duration of only 56.82 s and an average fixation duration of 0.15 s, indicating that this area received relatively little visual attention.

### 3.2 Correlation analysis of eye-tracking data and subjective ratings

#### 3.2.1 Analysis of total fixation duration for visual targets across six types of sites

**Figure 4:**
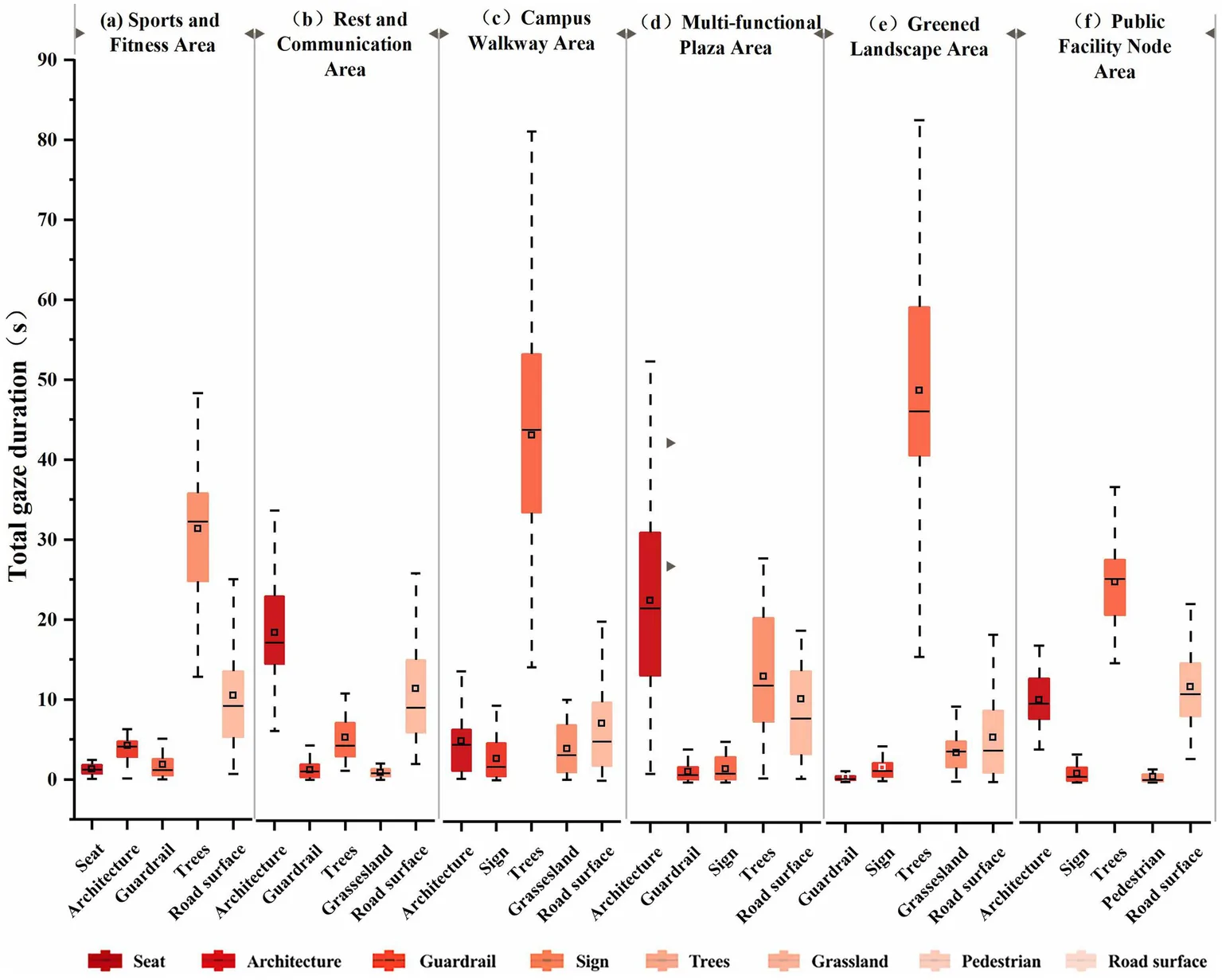
Total fixation duration of environmental elements in six types of sites. **(a)** sports and fitness area; **(b)** rest and communication area; **(c)** campus walkway area; **(d)** multi‑functional plaza area; **(e)** greened landscape area; **(f)** public facility node area.

Figure 4 shows the statistical results for the total fixation duration at each environmental factor across the six types of sites. The distribution of total fixation duration across the six types of settings reveals a highly consistent pattern of preference: trees > road surface > architecture > grassland > guardrail > sign > seat > pedestrian > pond > streetlight > stone table. In particular, trees dominate the visual landscape across all sites, with the total time spent gazing at trees in the campus walkway and greened landscape area reaching a peak, significantly higher than comparable metrics at other areas (Ou et al., 2026). The rest and communication area had the lowest total fixation duration, with a relative decrease in the proportion of trees and a corresponding increase in the proportion of facilities. The statistical correlation indicates that natural landscape elements are highly associated with long-duration visual fixation, while facility-dominated spaces tend to correspond to shorter and less concentrated visual fixation states (Kreiz et al., 2025). Meanwhile, the rest and communication area, with the lowest visual fixation duration, corresponded to the lowest subjective satisfaction score (3.58) in the questionnaire data.

#### 3.2.2 Analysis of average fixation duration for visual targets across six types of sites

**Figure 5:**
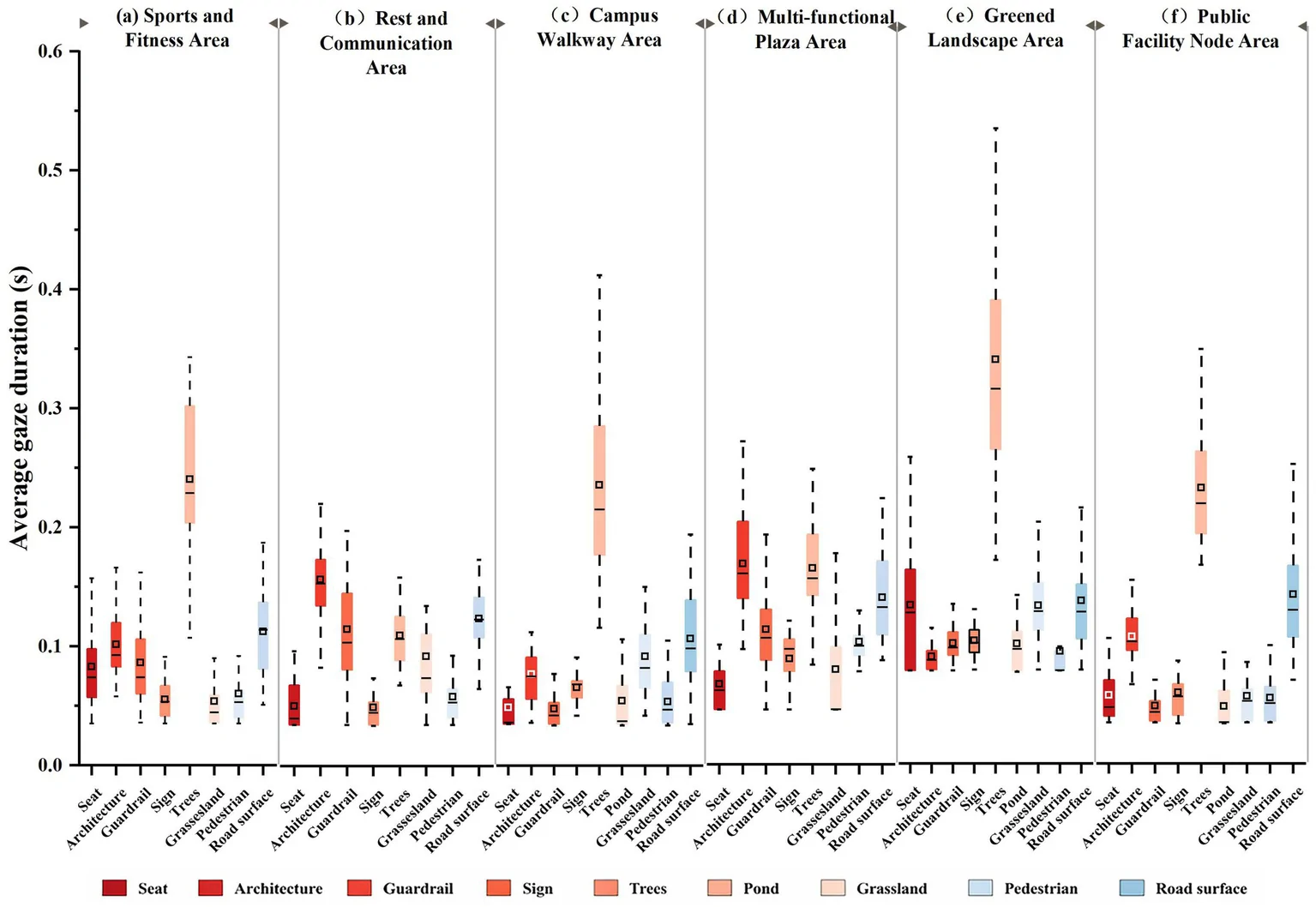
Average fixation duration on environmental elements in six types of sites. **(a)** sports and fitness area; **(b)** rest and communication area; **(c)** campus walkway area; **(d)** multi‑functional plaza area; **(e)** greened landscape area; (f) public facility node area.

Average fixation duration reflects the depth of information processing. The results in Figure 5 show that trees had the significantly highest average fixation duration, with a mean of 0.31 s, indicating that natural elements can elicit deep cognitive and emotional processing (Chen et al., 2022; Fleming et al., 2024). Road surface and sign followed, with average fixation durations of 0.11 s and 0.06 s, respectively, suggesting that these elements often involve safety assessments and information recognition tasks; facilities such as streetlight, stone table, and pond had the shortest average fixation durations, at less than 0.05 s, indicating shallow visual information processing and weak visual appeal. When analyzed by site type, the proportion of average fixation duration on road surface and sign in rest and communication area and public facility node area increased significantly. This indicates that such spaces suffer from poor legibility, blurred boundaries, and cluttered information, forcing participants to devote more cognitive resources to route confirmation and safety checks. This corresponds to the relatively low scores in the questionnaire for accessibility to recreational areas (2.53), auxiliary facilities (2.79), and shade and shelter (3.88). In contrast, campus walkway area and greened landscape area were characterized by high average fixation durations on trees, reflecting the low cognitive load and high relaxation provided by natural landscapes.

#### 3.2.3 Analysis of the first fixation duration on visual targets across six types of sites

**Figure 6:**
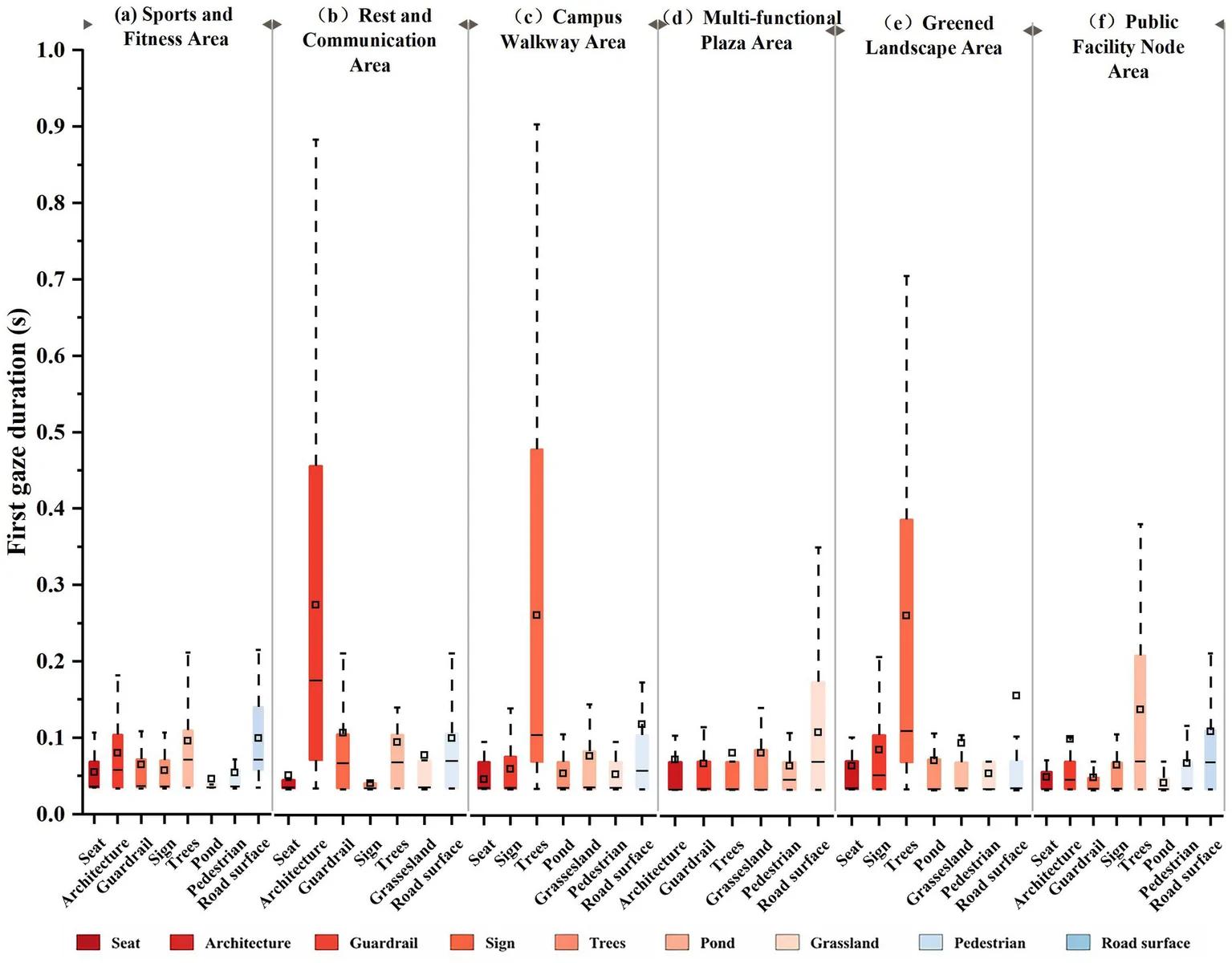
Analysis of first fixation duration of various environmental elements in six types of sites. **(a)** sports and fitness area; **(b)** rest and communication area; **(c)** campus walkway area; **(d)** multi‑functional plaza area; **(e)** greened landscape area; **(f)** public facility node area.

The first fixation duration reflects visual salience and the speed at which attention is captured. After participants arrive at the designated observation zone of a single site and real-time calibration of the equipment is completed, the system continuously records all fixation points starting from the 0th second upon entry into the site. The first stable fixation occurring within this site is defined as the first fixation on each element of the site. Figure 6 shows that trees, architecture, and road surface capture the first gaze the fastest, with durations significantly longer than other elements, indicating the strongest visual salience; facility-related elements (seat, streetlight, stone table) have shorter first fixation duration and weaker visual guidance. In terms of site differences, trees in the campus walkway area and the greened landscape area had the longest first fixation duration, indicating that natural elements serve as “strong visual anchors” in open environments; in contrast, the rest and communication area exhibited overall shorter and more scattered first fixation duration, lacking a stable focal point and demonstrating insufficient spatial distinctiveness. Combined with the eye-tracking questionnaire, participants found it more difficult to quickly locate seating in rest and communication area (average score 1.74) and to quickly identify signage in public facility node area (average score 1.74). This confirms that artificial facilities lack visual salience and blend too seamlessly into the environment, thereby affecting spatial efficiency and a sense of security (Wang et al., 2025a,b).

#### 3.2.4 Analysis of fixation count for visual targets in six types of sites

**Figure 7:**
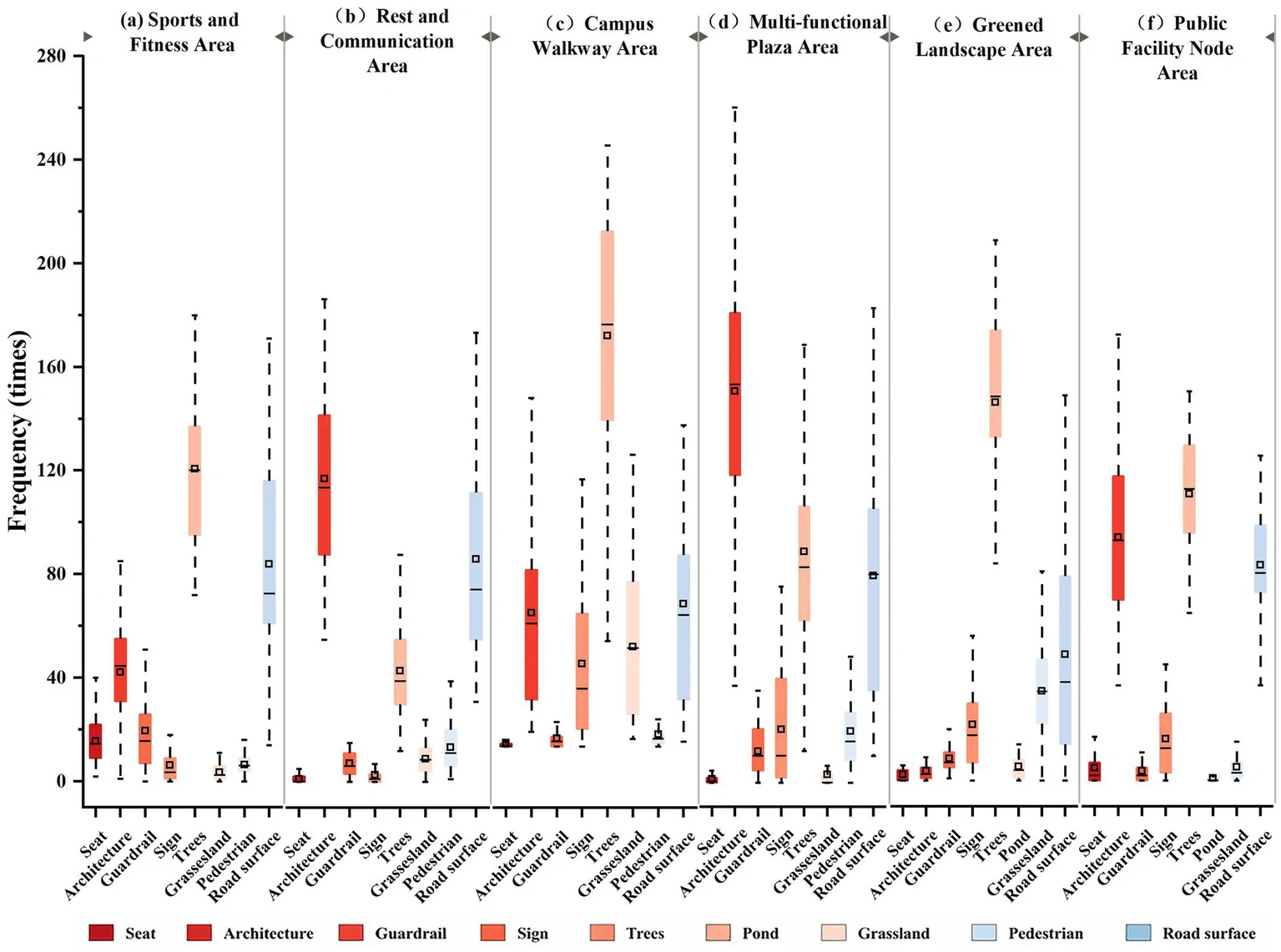
Analysis of the fixation count on various environmental elements in 6 types of sites. **(a)** sports and fitness area; **(b)** rest and communication area; **(c)** campus walkway area; **(d)** multi‑functional plaza area; **(e)** greened landscape area; **(f)** public facility node area.

The fixation count reflects the frequency and stability of visual attention. Figure 7 shows that trees receive far more gazes than other elements, exhibiting characteristics of high-frequency, repeated, and sustained attention; the campus walkway area and greened landscape area receive the highest fixation count, while the rest and communication area receives the lowest. This indicates that natural elements can sustain a steady flow of attention, whereas facility-dominated spaces struggle to retain visual attention. From a functional perspective, in the sports and fitness area, safety requirements led to a relative increase in fixation count toward guardrail and road surface; in multi-functional plaza area, gazes were concentrated on the boundaries of greenery and pedestrian paths, while gaze frequency was low in hard-surfaced activity zones; in public facility node area, due to the dense concentration of signage and complex ground level variations, the frequency of passive glances has increased; however, these are all instances of passive, scan-like glances rather than active engagement. Subjective data similarly reveals that the areas participants most wished to improve were concentrated in rest and communication area, sports and fitness area, and public facility node area—a finding that aligns closely with the areas exhibiting low gaze frequency and poor visual experience (Ghavampour, 2014).

#### 3.2.5 Analysis of total fixation duration and fixation count on visual targets

**Figure 8:**
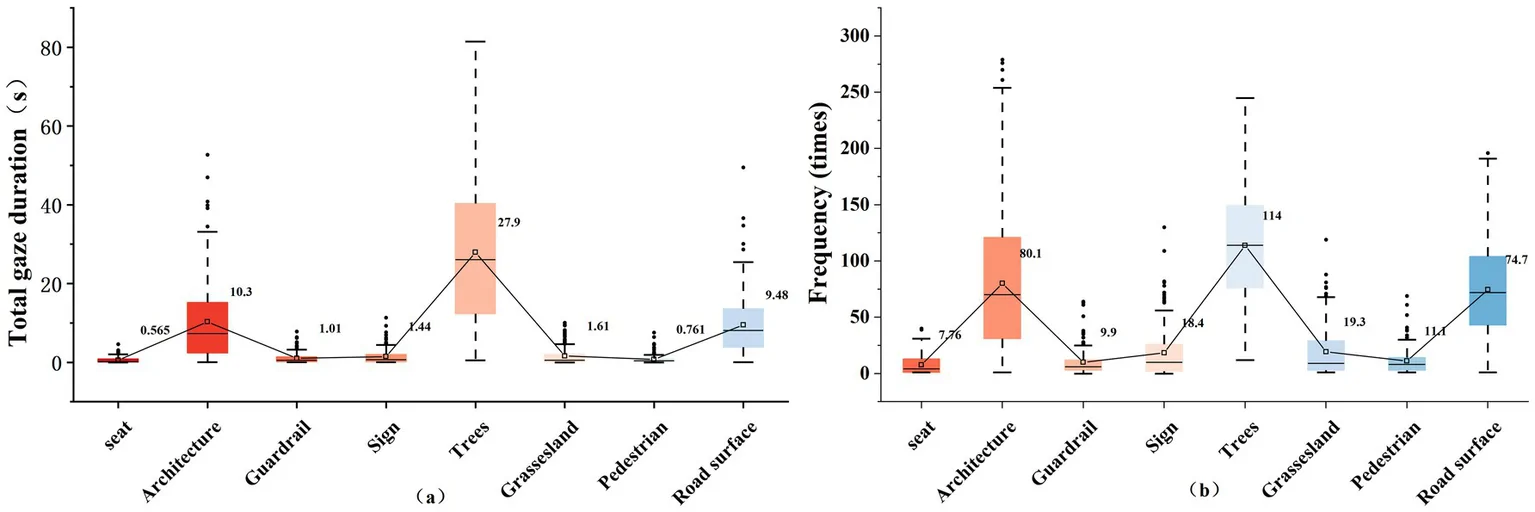
Analysis of the total gaze duration and number of gazes for all environmental elements.

Total fixation duration and fixation count show a very strong positive correlation (*r* = 0.99), indicating that female college students’ visual attention is primarily characterized by frequent, repetitive fixations rather than single, prolonged fixations. Figure 8 collectively demonstrates that trees rank first in both total fixation duration and fixation count, with natural elements dominating the scene (Weng et al., 2024). Road surface and architecture, as foundational elements, serve a spatial orientation function; facilities such as seat, sign, and streetlight receive significantly less attention. This pattern reveals that female college students in outdoor campus spaces are primarily drawn to natural landscapes and maintain a pleasant gaze toward them; they only direct compensatory gazes toward man-made elements when safety is uncertain, information is unclear, or facilities are difficult to locate. These findings provide crucial support for the mutual validation of subjective and objective data, and offer guidance for the design of gender-inclusive spaces.

#### 3.2.6 Analysis of eye-tracking heatmaps and gaze trajectory maps

As can be seen from the heatmap distribution characteristics of the six typical sites (see Table 1), greened landscape area and campus walkway area exhibit large-scale, high-intensity clusters of high heat, These high-heat cores are concentrated in tree canopies, expansive green spaces, and linear green pathways, indicating that female college students’ visual attention toward natural elements is sustained and focused. This finding is highly consistent with the results from quantitative eye-tracking metrics, which show that the total fixation duration and fixation count directed at trees are significantly higher than those for other elements (Liu et al., 2018; Ibes and Forestell, 2022; Fleming et al., 2024; Weng et al., 2024; Aghabozorgi et al., 2025).

**Table 1:** Heat map analysis of six types of sites.

Site type	Participant 1	Participant 2	Participant 3	Participant 4
(a) Sports and fitness area	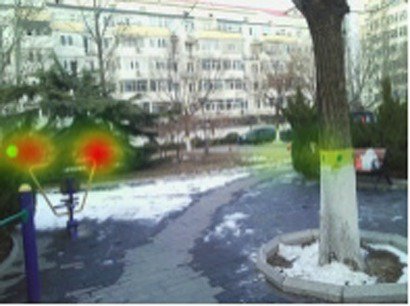	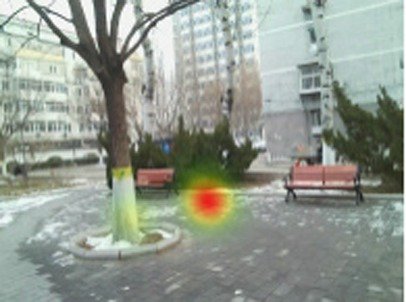	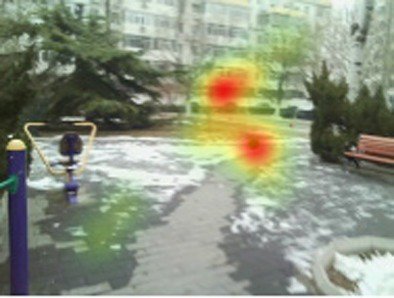	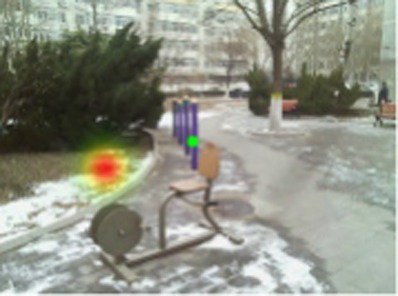
(b) Rest and communication area	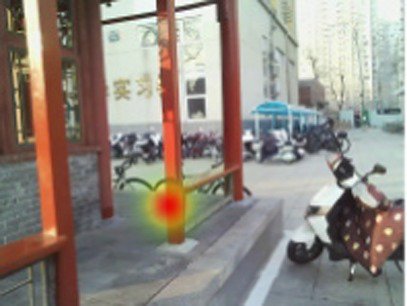	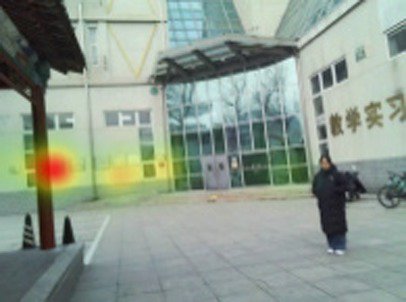	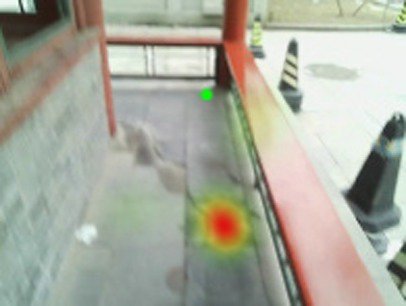	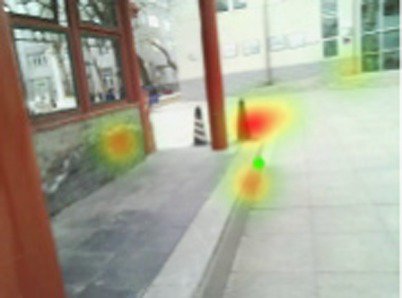
(c) Campus walkway area	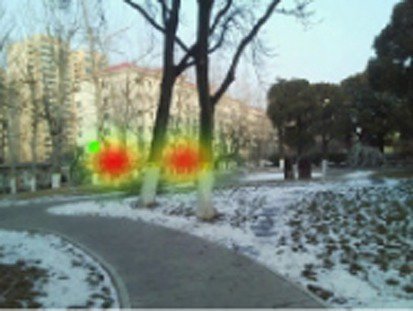	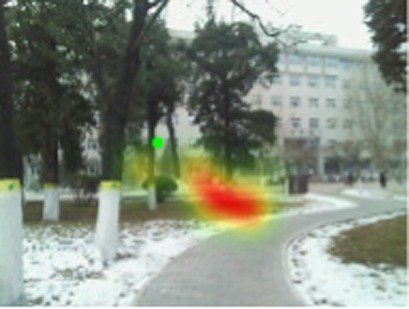	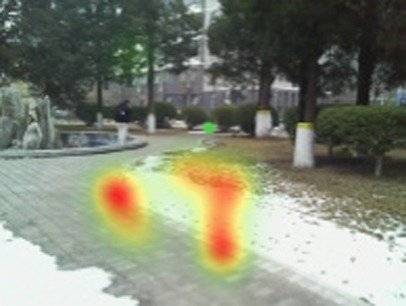	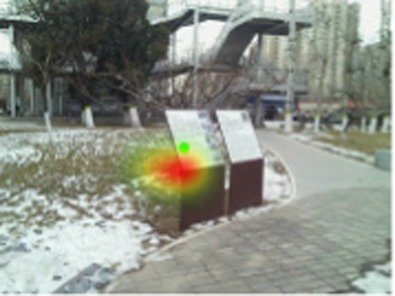
(d) Multi-functional plaza area	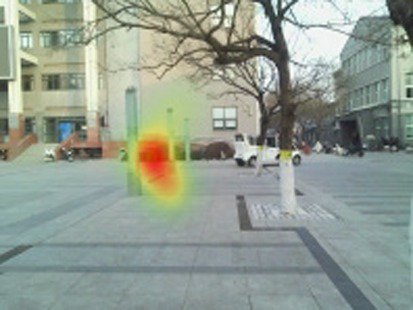	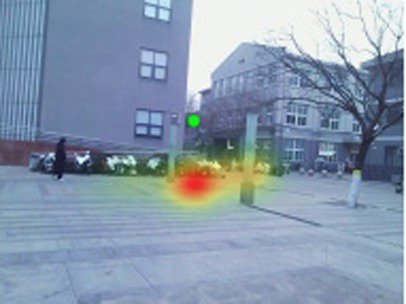	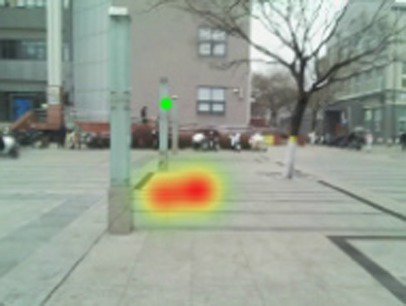	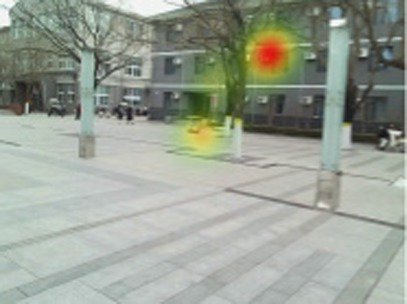
(e) Greened landscape area	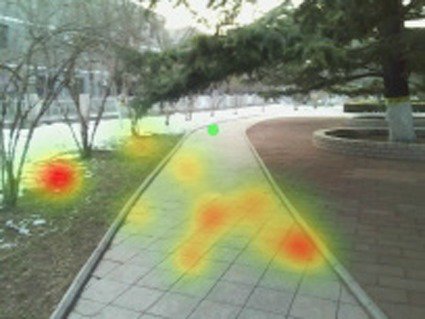	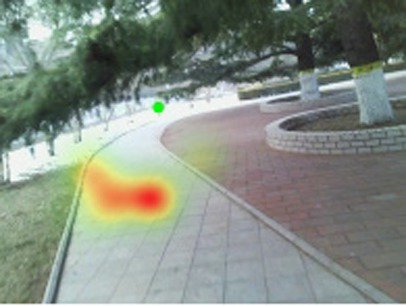	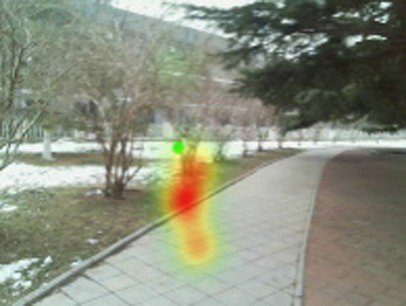	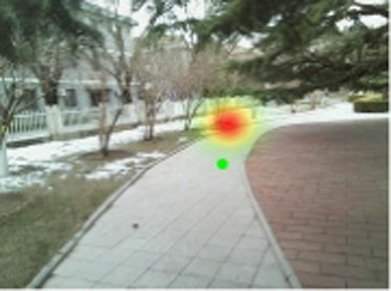
(f) Public facility node area	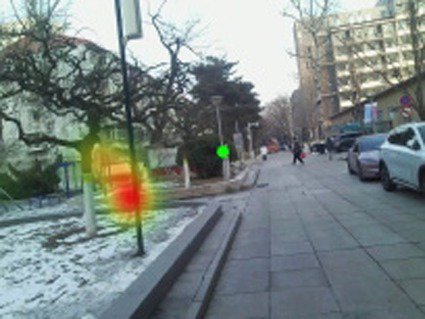	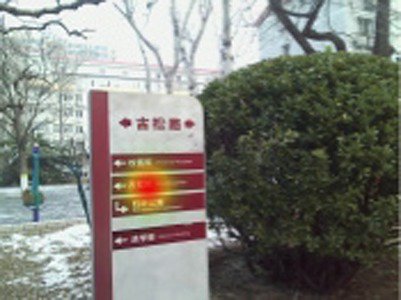	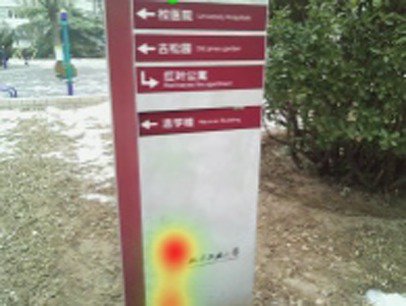	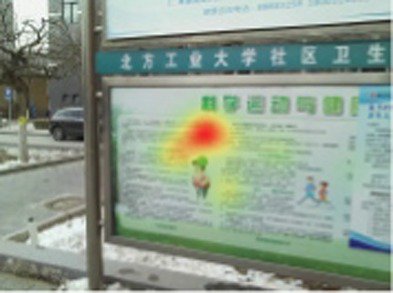

The multi-functional plaza area exhibits a dispersed, moderate level of activity concentration, with high heat maps concentrated along the edges of green spaces and pedestrian pathways. Hard surfaces and functional facilities show lower heat levels, reflecting a continued prioritization of natural landscapes within the open space. In the sports and fitness area, heat maps are concentrated around the surrounding greenery and safety barriers, while the fitness equipment itself shows lower heat levels, consistent with women’s heightened sensitivity to safety in public spaces (Sadeghi et al., 2023). The rest and communication area and public facility nodes area generally exhibit low heat maps with scattered distribution; only a few areas with seating and small potted plants show sporadic high heat, which visually corroborates the quantitative findings indicating low levels of visual attention and subjective satisfaction.

The results of the overall heatmap visualization are consistent with the eye-tracking metrics and subjective evaluations, clearly demonstrating that female college students’ visual attention is strongly drawn to elements of natural landscapes (Lu and Fu, 2019; Li et al., 2021; Jiang C. et al., 2025; Jiang G. et al., 2025; Zheng et al., 2025). The green coverage rate and the continuity of the natural landscape directly determine the intensity and stability of visual attention (Kang and Kim, 2019; Li et al., 2020). Spaces rich in natural elements are more likely to attract sustained visual attention, which not only corroborates the findings of similar studies but also provides objective evidence of visual behavior for optimizing outdoor spaces on campus (Lu and Fu, 2019; Wang et al., 2025a,b).

The gaze trajectories for each area are shown in Table 2. Trajectories in campus walkway area and greened landscape area are dense and continuous, with a strong focus on natural elements; in multi-functional plaza area, they are distributed along the greenery and pathways; in sports and fitness area, they tend to follow the guardrail and greenery; and in the leisure and facility areas, the trajectories are scattered and fragmented. Trajectory patterns are corroborated by eye-tracking and questionnaire data; natural elements determine the intensity of visual attention and the quality of the spatial experience (Evans and Chamberlain, 2024).

**Table 2:** Viewpoint trajectory diagrams for each site.

(a) Sports and fitness area	(b) Rest and communication area	(c) Campus walkway area
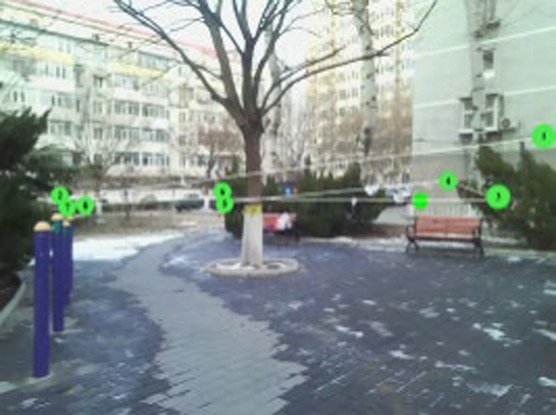	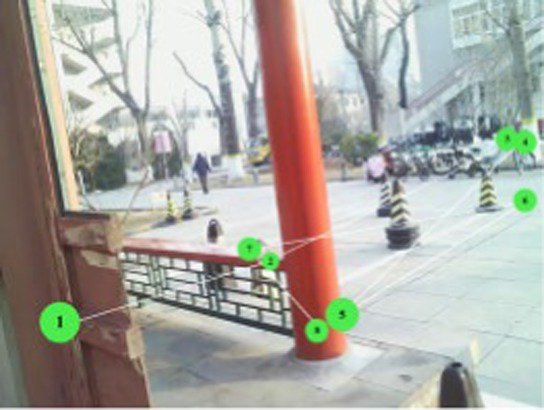	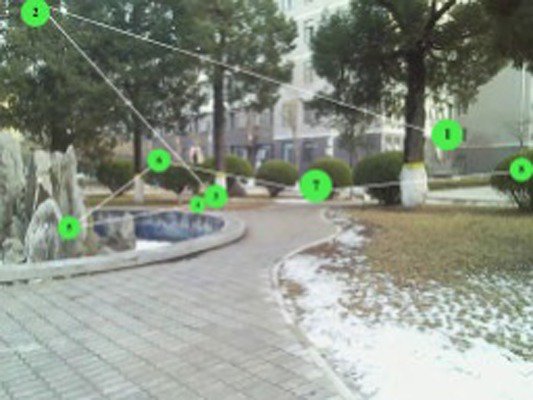
(d) Multi-functional plaza area	(e) Greened landscape area	(f) Public facility node area
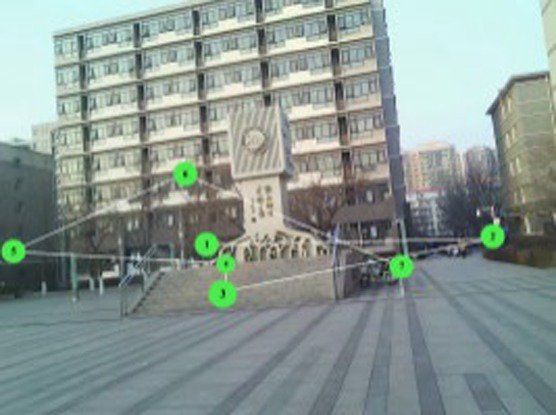	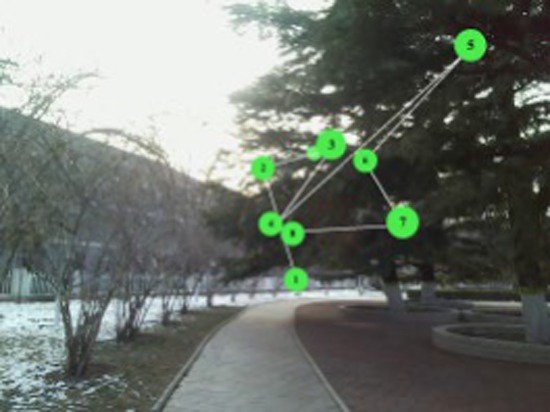	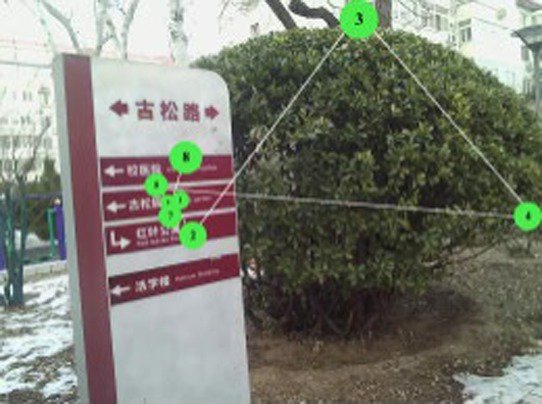

### 3.3 Correlation analysis of eye-tracking metrics

#### 3.3.1 Correlation analysis between eye-tracking metrics and overall satisfaction

Pearson correlation analysis was used to examine the relationship between eye-tracking metrics for 11 types of visual targets and overall satisfaction with each venue (see in Table 8 in Supplementary material), the results of the correlation analysis show that:

(1) Natural elements showed a significant positive correlation with satisfaction: all eye-tracking metrics related to trees exhibited a very strong positive correlation with overall satisfaction with the site (*r* = 0.482–0.683, *p* < 0.01); all eye-tracking metrics related to grassland showed a weak positive correlation with satisfaction (*r* = 0.276–0.328, *p* < 0.05), indicating that greater visual attention to natural elements is associated with higher spatial satisfaction (Zhang et al., 2022).
(2) Functional elements showed a significant negative correlation with satisfaction: eye-tracking metrics for road surface and sign exhibited a moderate negative correlation with satisfaction (*r* = −0.312 to −0.425, *p* < 0.01 or *p* < 0.05); The total fixation duration on guardrail showed a weak negative correlation with satisfaction (*r* = −0.269, *p* < 0.05), suggesting that excessive attention to functional elements such as road surface and sign may stem from concerns about safety hazards or information confusion, thereby reducing spatial satisfaction (Aziz, 2025).
(3) No significant correlation with other factors: There was no significant correlation (*p* > 0.05) between eye-tracking metrics for visual targets such as architecture, seat, and pedestrian and overall satisfaction, indicating that these factors have a relatively limited impact on overall satisfaction.

#### 3.3.2 Correlation analysis between eye-tracking metrics and 11 categories of visual targets

**Figure 9:**
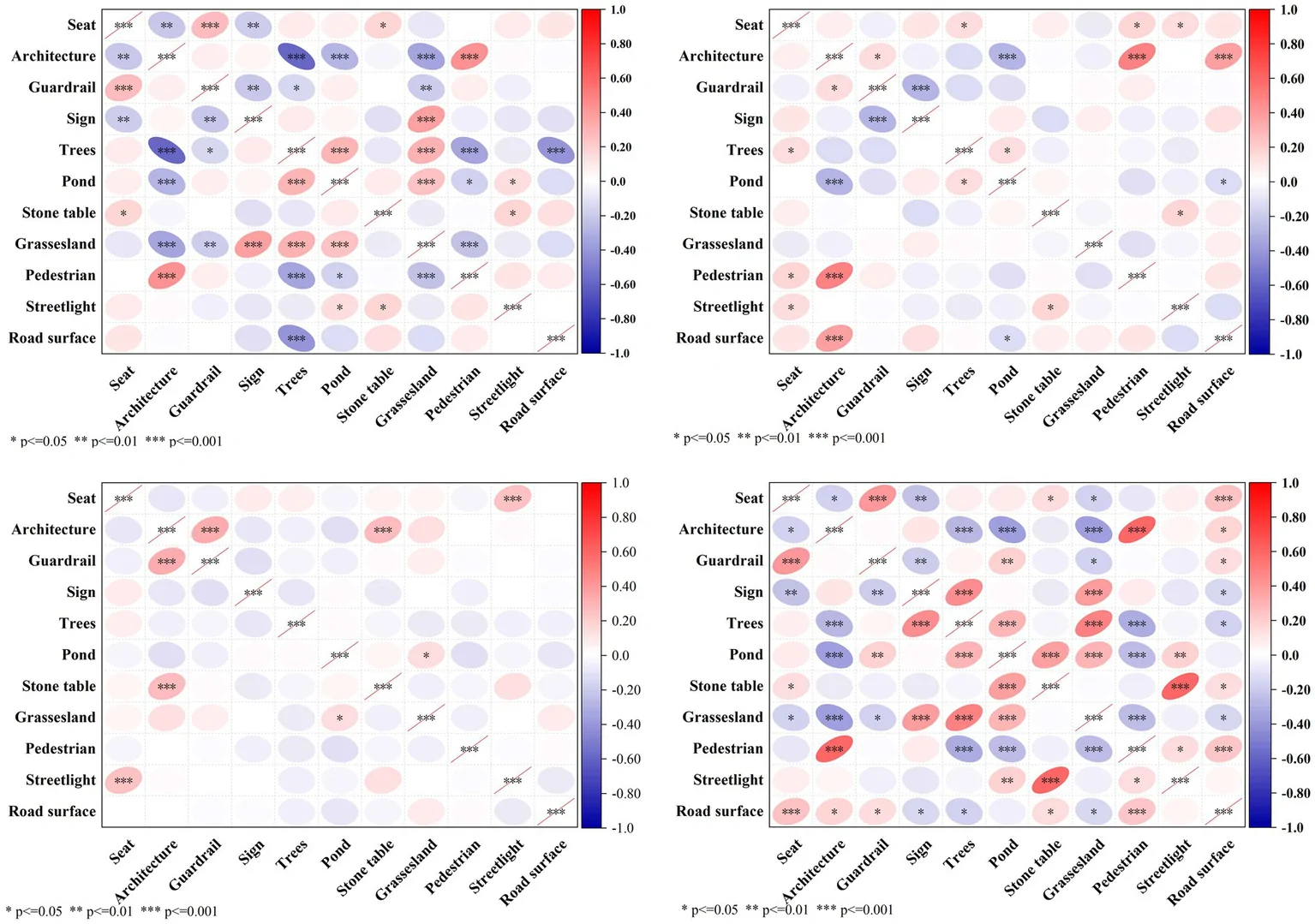
Analysis of the total fixation duration and fixation count for all environmental elements.

To investigate the visual attention characteristics of 11 types of spatial elements, a correlation analysis was conducted on four eye-tracking metrics: total fixation duration, average fixation duration, first fixation duration, and fixation count. This analysis revealed the patterns of visual attention distribution among different spatial elements and the underlying mechanisms driving this behavior. The results are shown in Figure 9.

The results of the correlation analysis show that:

(1) The correlations among the four eye-tracking metrics exhibit distinct hierarchical differences: first fixation duration is most strongly correlated with fixation count, followed by total fixation duration, while average fixation duration shows the weakest correlation. First fixation duration has a significant impact on the overall distribution of visual attention, whereas average fixation duration is relatively independent; it reflects only the user’s characteristics of single-instance information processing for a single environmental element and shows a lower correlation with other total eye-tracking metrics.
(2) Natural landscape elements such as trees, pond, and grassland are highly interrelated; their overall visual appeal works in concert to create a significant visual clustering effect. Users’ visual preferences for the natural environment stem from the combined influence of the overall ecological interface. Natural landscapes exhibit a significant negative correlation with man-made elements such as architecture and pedestrian; the two compete for visual attention. While natural landscapes emphasize sensory relaxation, man-made environments prioritize functionality and social interaction, resulting in a distribution of visual attention where one gains at the expense of the other (Zhou et al., 2022; Jiang C. et al., 2025; Jiang G. et al., 2025).
(3) The visual connections among elements within natural landscapes are strong, creating a prominent overall appeal; shifts in visual focus tend to occur simultaneously, and users’ perception of the natural environment relies on the overall ecological atmosphere rather than individual features. There is significant visual competition between natural landscapes and man-made facilities or human activities; the visual perception of these two types of environments is directed toward different goals, and the allocation of attention is mutually constrained (Fei et al., 2022).
(4) As key elements driving the vitality of a space, architecture and pedestrian exhibit highly consistent patterns of eye movement, and together with the natural landscape, they form the two primary visual focal points of the public space. The building facades and pedestrian activity are interrelated, jointly creating a sense of place, and complement the ecological visual experience provided by the natural landscape (Misthos et al., 2023).

In summary, the patterns of visual attention in public spaces are jointly influenced by the visual characteristics of elements, spatial functional layout, and the types of elements. These three factors, respectively, determine attention bias, the association patterns of man-made facilities, and differences in the distribution of attention between natural and man-made elements, thereby providing empirical support for gender-responsive spatial design and landscape optimization (Suh and Cho, 2021).

## 4 Discussion

### 4.1 Visual behavior characteristics of female college students

This study indicates that female college students exhibit typical gender-specific characteristics in their visual behavior within outdoor campus spaces, including strong selectivity, high concentration, and a safety-oriented focus; their allocation of visual attention is not random but reflects a stable and adaptive cognitive pattern (Jiang C. et al., 2025; Jiang G. et al., 2025). First, they are highly sensitive to potential risks and pay particular attention to road surface smoothness, the clarity of boundaries, elevation differences, and the stability of infrastructure (Huang et al., 2022). Survey data shows that the proportion of people frequently looking down in sports and fitness area due to concerns about slippery floors (0.88), the proportion looking down in campus walkway area due to surface issues (0.97), and the proportion paying attention to minor elevation changes in public facility node area (0.94) indicate that visual behavior directly serves the purpose of risk assessment. Second, visual processing depends on the overall legibility and controllability of a space: in open, clearly defined spaces with appropriate enclosure, gaze patterns remain stable; whereas in spaces with severe obstructions and cluttered information, visual scanning becomes fragmented and exhibits anxiety-related characteristics (Meneghetti et al., 2017). Finally, female college students showed a clear preference for natural landscapes, with the proportion of total fixation duration directed at natural elements such as trees (0.566) indicating that natural landscapes serve not only as visual objects but also play a role in emotional regulation and stress relief (He et al., 2024; Liu et al., 2025; Pullman, 2025; Zhang et al., 2026). Furthermore, women rely more heavily on the integration of visual information during spatial cognition, which makes them more prone to developing path dependence and spatial preferences in complex environments (Dahmani et al., 2023). In summary, their visual behaviors are driven by a combination of safety, cognitive, and emotional needs, and can provide both theoretical foundations and empirical evidence for gender-responsive campus space design (Chaney et al., 2024).

### 4.2 The influence of visual attention to spatial elements on spatial satisfaction

The results of this study indicate that natural landscapes (particularly trees) dominate visual attention, with significantly higher total fixation duration, average fixation duration, and fixation count compared to other elements. Further analysis revealed that attention to trees was significantly positively correlated with spatial satisfaction (*r* = 0.726, *p* < 0.001), and attention to grassy areas was also significantly positively correlated with spatial satisfaction (*r* = 0.583, *p* = 0.001), consistent with existing research in environmental psychology (Wang et al., 2025a,b). Numerous studies have shown that natural landscapes can significantly enhance the quality of the environmental experience through visual appeal and psychological restoration mechanisms (DeLauer et al., 2022; Liu et al., 2022; Wang et al., 2024; Jiang C. et al., 2025; Jiang G. et al., 2025). Natural elements typically feature high visual complexity and a gentle visual structure, making them more likely to sustain attention and thereby promote emotional recovery and mental relaxation. In studies of campus environments, green spaces are considered a key factor influencing college students’ mental health and satisfaction with their surroundings (Liu et al., 2022). The positive impact of natural landscapes on college students’ mental health stems not only from the aesthetic appeal of the landscapes themselves, but also from their buffering effect on cognitive fatigue and emotional stress (Lu and Fu, 2019). The eye-tracking data from this study further corroborate this conclusion from the perspective of objective visual behavior. For female college students, natural landscapes not only provide visual comfort but also enhance a sense of spatial security and emotional stability; this may be related to the fact that women tend to seek cues of safety and comfort in their perception of the environment (He et al., 2024; Pullman, 2025; Zheng et al., 2025). Furthermore, results for different types of spaces indicate that greened landscape area and campus walkway area scored significantly higher on eye-tracking metrics than other types of spaces, and also ranked relatively higher in subjective satisfaction evaluations (4.19, 4.12). This suggests that the combination of natural landscapes and linear pedestrian spaces creates a positive visual experience, thereby enhancing the overall evaluation of the space (Li et al., 2024). Therefore, in the planning of outdoor spaces on campus, greater emphasis should be placed on enhancing the visual accessibility and spatial integration of natural landscape elements—for example, by increasing tree shade, optimizing the layout of green spaces, and enhancing landscape layering—to improve the visual appeal and user experience of these spaces (Lau et al., 2014).

In addition, there was a significant negative correlation between eye-tracking metrics for functional elements such as road surfaces and traffic signs and spatial satisfaction (*r* = −0.674, *p* < 0.001; *r* = −0.512, *p* = 0.002). In other words, when participants directed more visual attention toward these elements, their overall spatial satisfaction was lower (Kim and Park, 2020; Liu et al., 2023). At the same time, in the low-satisfaction group, the fixation count and the duration of fixations on the road surface and sign were significantly higher than in the high-satisfaction group. This phenomenon can be explained by environmental cognition theory. When a spatial environment presents potential uncertainty or cognitive difficulties, individuals often need to devote more visual attention to processing environmental information (Orquin et al., 2021). For example, when there are changes in slope, steps, or potential safety hazards on the ground, users increase their visual monitoring of the ground, resulting in more frequent and longer fixations (Hasanzadeh et al., 2018). Similarly, when wayfinding systems are unclear or lack sufficient information, individuals must repeatedly examine the signs to understand the spatial information, thereby increasing their visual attention to the signs (Iftikhar et al., 2021). Related studies indicate that spatial legibility is a key factor influencing the experience of public spaces (Iftikhar et al., 2021). A well-designed wayfinding system and a clear spatial structure can reduce cognitive load, thereby enhancing environmental comfort (Costa Bomfim and Santos Cruz, 2023). In studies on women’s sense of safety, researchers have also noted that when spatial information is unclear or pathways are complex, women are more likely to experience feelings of insecurity or anxiety (Dubey et al., 2025).

In this study, subjective evaluations of public facility node area and rest and communication area revealed deficiencies in wayfinding information and accessibility features. Eye-tracking data also showed a high level of attention directed toward signage and pavement surfaces, indicating consistency between subjective evaluations and objective visual behavior. Therefore, optimizing outdoor spaces on campus requires not only a focus on landscape quality but also improvements in the clarity and usability of functional facilities (Elbertsen et al., 2025).

### 4.3 The coupling relationship between visual behavior and environmental safety

This study indicates that female college students’ visual behavior is closely linked to their sense of environmental safety and can serve as an objective indicator of safety (Lu and Pesarakli, 2023; Jiang C. et al., 2025; Jiang G. et al., 2025; Wang et al., 2025a,b). In high-security spaces, users’ gaze is primarily focused on trees and greenery; their movements are slow and deliberate, with a fixed field of view. The spaces are open and airy, with clearly defined boundaries, resulting in higher subjective safety ratings (Tabatabaie et al., 2023). Conversely, in areas with low perceived safety or complex environments, factors such as uneven pavement, unclear signage, and obstructed views cause frequent shifts in gaze and fragmented attention. Eye-tracking data shows a significant increase in fixations on the pavement and signage (2.53 in rest and communication area, 2.65 in sports and fitness area, and 1.85 in public facility node area), leading to a decline in perceived safety and comfort. According to theories in environmental psychology and neuroarchitecture, female college students prefer spaces with open views, stable boundaries, and natural vegetation for screening. In such environments, users experience a more stable visual state, lower levels of psychological anxiety, and a stronger desire to linger in the space (Akcelik et al., 2024). From the perspective of gendered spatial behavior, women’s visual behavior in public spaces typically exhibits more pronounced defensive cognitive characteristics (Navarrete-Hernandez et al., 2021). Spaces with limited visibility, poorly defined pathways, and inadequate amenities tend to trigger a state of visual vigilance in users, thereby reducing their willingness to actually use them (Han et al., 2022; Palaneer and Sharmin, 2026). Correlation analysis further confirms that excessive fixation on road surface, sign, and guardrail is significantly negatively correlated with a sense of safety (*r* = −0.701, *p* < 0.001, *r* = −0.547, *p* = 0.001, *r* = −0.489, *p* = 0.003), while fixation on natural elements is significantly positively correlated with a sense of safety (*r* = 0.695, *p* < 0.001, *r* = 0.538, *p* = 0.001) (Li et al., 2024). As can be seen, visual behavior is not merely the result of perception, but also a prerequisite for safety decisions; optimizing spatial visual cues helps enhance women’s sense of security (Maloney and Zhang, 2010).

### 4.4 Cognitive explanations for discrepancies between subjective and objective evaluations

This study found that while there were some local discrepancies between eye-tracking data and subjective ratings, the two were generally consistent, reflecting a hierarchical mechanism involving unconscious physiological responses and conscious rational judgments (Glöckner et al., 2012; Purcell et al., 2022; Bortolotti and Palumbo, 2026). Eye-tracking data reflects immediate, automatic attentional preferences and provides a more accurate picture of visual appeal and emotional responses; for example, the high level of gaze directed at trees in greened landscape area and along campus walkway area is driven by instinctive pleasure (Zhu and Lv, 2023). Subjective ratings, however, are influenced by factors such as functionality, the completeness of facilities, and overall impression, and may therefore be higher than what one might intuitively expect. Subjective evaluations take into account factors such as functionality, cleanliness, management, and convenience; consequently, even if the natural scenery is excellent, inadequate facilities can still lower overall satisfaction—as evidenced by the overall rating for rest and communication area (3.58 points) (Johnson, 2025). When spatial information is cluttered, signage is unclear, or road conditions are complex, participants experience a high objective visual load; however, their subjective ratings may not reflect extremely low scores due to habituation, resulting in a latent discrepancy (Minkley et al., 2021; Nordmark, 2025). This discrepancy suggests that spatial experiences are not determined solely by visual appeal, but rather by the combined effects of environmental aesthetics, functional convenience, a sense of security, and cognitive load. Compared to simple questionnaire surveys, eye-tracking provides a more accurate picture of subconscious visual preferences and sources of environmental stress (Chen et al., 2023). Furthermore, this study combined semantic segmentation with eye-tracking mapping technology to establish a precise correspondence between visual attention and spatial elements, thereby enhancing the objectivity and ecological validity of research on spatial behavior in real-world settings. This also demonstrates that the integration of eye-tracking technology with artificial intelligence recognition technology offers a new research approach for evaluating campus spaces (Sundstedt and Garro, 2022). Therefore, combining eye-tracking with questionnaires can provide a more comprehensive understanding of spatial experiences and enhance the reliability of conclusions.

In the future, with advancements in VR, digital twin campuses, mobile physiological sensors, and AI-based environmental recognition technologies, research on campus spaces will gradually shift from “static preference assessment” to “dynamic cognitive-behavioral modeling,” thereby enabling real-time prediction and precise optimization of spatial experiences (Alnaser et al., 2024).

### 4.5 Correlation between element spatial proportion and visual attention

Combining the heatmaps in Table 1 and gaze trajectory diagrams in Table 2 of this study, it can be intuitively observed that trees, green plants and other natural landscapes occupy the largest continuous areas within the frame in most experimental scenes. This advantage in visual field area objectively raises the probability that gaze points randomly fall on vegetation zones, acting as a confounding factor that cannot be ignored when interpreting eye-tracking fixation indicators (Evans and Chamberlain, 2024).

According to the original eye-tracking statistical results, trees score significantly higher across all four core metrics—total fixation duration, average fixation duration, first fixation duration and fixation count—than artificial hardscape elements such as pavements and buildings. Nevertheless, this outcome cannot be fully attributed to the inherent visual appeal of vegetation alone. From the perspective of frame composition, road surface and architecture also occupy considerable proportions of the visual field, yet all their eye-tracking metrics remain markedly lower than those of vegetation. This indirectly indicates that female participants allocate more visual resources to natural landscapes voluntarily, even after accounting for the passive gaze probability brought by the large visual coverage of greenery (Ma et al., 2023; Jiang C. et al., 2025; Jiang G. et al., 2025).

### 4.6 Limitations of the study and directions for future research

Although this study conducted a relatively systematic analysis of the visual behavior of female college students in outdoor campus spaces by combining eye-tracking technology with subjective evaluation methods, certain limitations remain. First, the sample size of this study is relatively small, comprising only 34 participants, and it primarily consists of first- and second-year students aged 18–20; thus, there is room for improvement in the representativeness of the sample. Future research could expand the sample size and include students from different academic years and disciplinary backgrounds to enhance the generalizability of the findings. Second, the experimental setting was limited to a single campus environment. Although the spatial typology was representative, it could not fully reflect spatial differences across regions or campus configurations. Future research could conduct cross-campus or cross-city comparative studies to explore variations in visual behavior across different cultural and spatial contexts. Third, landscape elements’ pixel proportion in the visual field creates confounding interference in eye-tracking interpretation. Vegetation dominates most frames in Tables 1, 2, raising the chance of random gaze hits. This study failed to perform AOI pixel normalization to distinguish passive area-induced fixations from preference-driven active gazes; future studies should calculate AOI pixel areas and adopt normalized eye-tracking metrics to eliminate such bias. In addition, all field experiments were conducted in Beijing’s winter (December 2025). At this time, most deciduous trees lose leaves, the overall vegetation coverage of campus outdoor spaces decreases, and the visual texture and color richness of green landscapes are significantly weaker than spring and summer conditions. The seasonal state of vegetation will inevitably interfere with female students’ visual capture and spatial cognitive responses to natural elements. In spring and summer, lush foliage, colorful flowers and layered plant landscapes may generate stronger visual salience and longer normalized fixation durations; in autumn, seasonal leaf color changes will also alter the distribution of visual attention. Since this study only collected winter data, the conclusions regarding the visual preference for natural elements cannot be fully generalized to all seasonal conditions. Subsequent comparative seasonal experiments across spring, summer, autumn and winter are required to clarify how vegetation seasonal variation modulates the visual behavior and spatial satisfaction of female college students. Furthermore, this study primarily focused on visual behavior under daytime conditions, whereas factors such as lighting conditions and perceived safety in nighttime environments also significantly influence women’s spatial experiences. Future research could combine nighttime environmental experiments with emotional measurement methods (such as physiological signals or emotional scales) to further deepen our understanding of campus spatial experiences.

## 5 Conclusion

This study focuses on female college students and adopts a mixed research framework integrating wearable eye-tracking equipment and subjective questionnaire surveys to carry out empirical investigation and systematic descriptive analysis of their visual behavioral patterns and spatial cognitive tendencies within campus outdoor activity spaces. By cross-verifying eye-tracking indicators and questionnaire feedback, this study systematically describes the regularities of visual attention distribution and spatial cognitive features of female college students in campus outdoor activity spaces, and summarizes corresponding quantitative statistical results without inferring causal interaction relations between variables.

In terms of visual fixation distribution, trees show the highest visual attention value among all natural landscape elements; indicators including total fixation duration, average fixation duration and fixation count are all markedly higher than those of other visual targets, which reflects a strong correspondence between natural vegetation and participants’ concentrated visual gaze. In terms of spatial types, campus walkways and green landscape area correspond to higher visual attention values among participants, whereas rest and communication area are associated with relatively lower visual fixation intensity. In subjective questionnaire scoring, multi-functional plaza area and campus walkway area are matched with the highest satisfaction scores reported by respondents, while rest and communication area correspond to the lowest satisfaction scores. Correlation analysis results show that visual attention toward natural elements is significantly positively correlated with spatial satisfaction, whereas visual attention toward functional elements such as road surface and sign is significantly negatively correlated with satisfaction. This correlation feature reflects a synchronous trend: spaces with complicated spatial identification conditions or perceived safety risks tend to be accompanied by higher visual fixation on pavement and signage elements, and such high fixation values are synchronously paired with lower subjective evaluation scores from participants. The joint sorting of eye-tracking indicators and questionnaire data identifies four dimensions that show obvious correlational linkage with female students’ outdoor space experience scores on campus: natural landscape conditions, spatial recognizability, facility comfort performance and space safety layout.

Notably, all visual behavior data in this study were captured under Beijing’s winter vegetation conditions, and the correlation characteristics between natural elements and visual attention may present seasonal variations that require further multi-season verification. Overall, this study describes the spatial perception features of female college students in campus outdoor spaces from the dual dimensions of visual behavior and environmental cognition, and supplies new empirical descriptive data for further exploring the spatial demand characteristics of female users. The correlational results obtained in this study can provide referential descriptive clues for the layout adjustment of campus outdoor public spaces, including enriching natural landscape configurations, sorting out navigation signage systems, upgrading facility comfort performance and adjusting space safety layouts.

## Data Availability

The raw data supporting the conclusions of this article will be made available by the authors, without undue reservation.

## Footnotes

1
https://github.com/facebookresearch/Mask2Former
