# Supplementary material for: A study on the visual behavior and cognitive preferences of female college students in outdoor campus spaces based on eye-tracking
Source: Front Psychol. 2026 Sep 3;17:1896634. doi: 10.3389/fpsyg.2026.1896634 (PMC13581501; doi:10.3389/fpsyg.2026.1896634)
Supplement: Supplementary file 1 [file Supplementary_File_1.docx]

**Appendix 1:**

**Table 1.** Key Technical Specifications of the aSeeglasses Eye Tracker

| **Parameter** | **Specific specifications** |
| --- | --- |
| Sampling rate | 60Hz / 120Hz / 240Hz |
| Accuracy | 0.5° |
| Delay | < 10ms |
| Audio | Built-in microphone |
| Calibration Method | Card calibration or physical calibration |
| Technology | Pupillary corneal reflex |
| Eyeglass size | ≤ 173 × 58 × 156 mm |
| Weight of glasses | 50g ± 3g |

**Table 2．**Definition of eye movement indicators

| **Indicator** | **Unit** | **Description** |
| --- | --- | --- |
| Total Fixation Duration**（TFD）** | s | Sum of all fixation durations on a target, indicating the overall attention intensity. |
| Average Fixation Duration**（AFD）** | s | Mean duration of individual fixations, reflecting the depth of information processing. |
| First Fixation Duration**（FFD）** | s | Duration of the initial fixation on a target, measuring early visual salience and initial cognitive engagement. |
| Fixation Count**（FC）** | time | Total number of fixations on a target, representing the frequency of visual attention. |

**Table 3．**Overall Statistics on Eye-Tracking Metrics for 11 Types of Visual Targets

| **Visual Objectives** | Total Fixation Duration (S) Mean ± Standard Deviation | Average Fixation Duration (S) Mean ± standard deviation | First Fixation Duration (S) Mean ± Standard Deviation | Fixation Count (times) Mean ± Standard Deviation |
| --- | --- | --- | --- | --- |
| pedestrians | 0.52±0.68 | 0.05±0.02 | 0.06±0.04 | 10.35±8.72 |
| road surface | 8.76±5.21 | 0.11±0.03 | 0.13±0.08 | 82.18±41.36 |
| trees | 34.29±15.76 | 0.31±0.14 | 0.23±0.16 | 118.47±56.89 |
| grassland | 0.87±1.12 | 0.07±0.03 | 0.08±0.05 | 13.24±12.81 |
| streetlights | 0.02±0.08 | 0.04±0.02 | 0.05±0.03 | 0.53±2.17 |
| architecture | 10.63±6.48 | 0.11±0.04 | 0.15±0.11 | 98.76±53.24 |
| seats | 0.82±0.94 | 0.08±0.04 | 0.08±0.06 | 10.59±9.87 |
| pond | 0.15±0.32 | 0.05±0.03 | 0.06±0.04 | 3.12±6.74 |
| stone tables | 0.01±0.05 | 0.04±0.02 | 0.04±0.02 | 0.29±1.21 |
| guardrails | 1.15±1.43 | 0.08±0.03 | 0.09±0.05 | 15.29±13.68 |
| sign | 1.21±1.57 | 0.06±0.02 | 0.07±0.04 | 21.03±18.96 |

**Table 4．**One-way ANOVA of eye-tracking metrics for 11 types of visual targets

| **Eye-tracking metrics** | **degree of freedom** | **F-value** | **P-value** | **Groups with significant differences in post-hoc tests (LSD)** |
| --- | --- | --- | --- | --- |
| Total Fixation Duration**（TFD）** | 10, 363 | 128.74 | <0.001 | Trees > Architecture > Pavement > Other objects; streetlights, stone tables, and fountains are significantly lower than other objects |
| Average Fixation Duration**（AFD）** | 10, 363 | 47.32 | <0.001 | Trees > Buildings, Pavements > Pedestrians, signs, streetlights, etc.; stone tables, fountains, pedestrians are noticeably lower |
| First Fixation Duration**（FFD）** | 10, 363 | 28.56 | <0.001 | Trees > Architecture > Roadways > Other objects; streetlights and stone tables are significantly lower than other objects |
| Fixation Count**（FC）** | 10, 363 | 89.45 | <0.001 | Trees > Architecture > Pavement > Signs > Guardrails > Other objects; streetlights, stone tables, and fountains are significantly less common |

**Appendix 2:**

**Questionnaire on A Study on the Visual Behavior and Cognitive Preferences of Female College Students in Outdoor Campus Spaces Based on Eye-Tracking**

**Dear Participants,**

Thank you for participating in this on-site experience. To help us better understand your genuine impressions of the outdoor activity areas on campus, please evaluate the following descriptions based on what you just observed and experienced firsthand at the site. Please check the box (“√”) next to the option that best reflects your feelings. All your responses will be kept confidential and used solely for academic research.

**I. Basic Information**

**1. Participant's Name： ______**

**2. Venue Number/Name of the venue you visited today：______**

**3. Your age：**

**□ Ages 18–20**

**□ Ages 21–23**

**□ Ages 24–25**

**4. Your gender：**

**□ Male**

**□ Female**

**5. Your vision (including corrected vision)：**

**□ Clear and normal**

**□ Mild blurriness**

**□ Moderate blurriness**

**□ Severe blurriness**

**6. How often do you participate in outdoor activities on campus each week?：**

**□ Almost every day**

**□ 3–5 times**

**□ 1–2 times**

**□ < 1 time**

**7. The times when you are usually active：（Multiple selections allowed）**

**□ Morning (6–9 a.m.)**

**□ Morning (9 a.m.–12 p.m.)**

**□ Afternoon (12:00–17:00)**

**□ Evening (5:00–7:00 p.m.)**

**□ At night (after 7:00 p.m.)**

8. **What is your main purpose for using outdoor spaces?：（Multiple selections allowed）**

**□ Sports and Fitness**

**□ Social Conversation**

**□ Resting/Relaxing Alone**

**□ Study/Reading**

**□ Other _____**

**II. Site-by-Site Assessment**

1. **Sports and Fitness Area**

**（1） Subjective Satisfaction Questionnaire (**Scoring Guide: 1 = Very Dissatisfied, 2 = Somewhat Dissatisfied, 3 = Neutral, 4 = Somewhat Satisfied, 5 = Very Satisfied**)**

| **No.** | **Review Content** | **1point** | **2points** | **3points** | **4points** | **5points** |
| --- | --- | --- | --- | --- | --- | --- |
| **1** | **Safety of the equipment (presence of sharp edges or corners, stability)** | **□** | **□** | **□** | **□** | **□** |
| **2** | **Readability of Signs and Instructions (font size, clarity, ease of understanding)** | **□** | **□** | **□** | **□** | **□** |
| **3** | **Are the seats arranged in a way that makes it easy to rest between exercises, and are they positioned so that you can see your surroundings?** | **□** | **□** | **□** | **□** | **□** |
| **4** | **The slip resistance, levelness, and shock absorption of floor materials (e.g., risk of falling)** | **□** | **□** | **□** | **□** | **□** |
| **5** | **Is the contrast between the equipment's color and the background sufficient to make it easily recognizable from a distance?** | **□** | **□** | **□** | **□** | **□** |
| **6** | **Do the operating instructions include both text and illustrations, and are the steps simple and clear?** | **□** | **□** | **□** | **□** | **□** |
| **7** | **Is there sufficient spacing between the equipment to prevent interference during use?** | **□** | **□** | **□** | **□** | **□** |
| **8** | **Do the sunshades and rain shelters cover some of the equipment, making it easier to use in different weather conditions?** | **□** | **□** | **□** | **□** | **□** |
| **9** | **Quietness of the environment: Is it located away from traffic or noisy areas, making it easy to focus on your workout?** | **□** | **□** | **□** | **□** | **□** |
| **10** | **Social atmosphere: Does it make it easy to interact with others or observe them exercising, thereby increasing the desire to participate?** | **□** | **□** | **□** | **□** | **□** |
| **11** | **Overall aesthetic appeal; whether the equipment blends harmoniously with the surrounding environment and creates a pleasant atmosphere** | **□** | **□** | **□** | **□** | **□** |
| **12** | **Adaptability: Are there equipment options suitable for different physical conditions (such as strength and balance)?** | **□** | **□** | **□** | **□** | **□** |

**（2）Eye Movement Correlation Questionnaire (Each question has equidistant quantitative response options, such as “Never,” “Occasionally,” “Often,” and “Always,” corresponding to scores of 1–4)**

| **AOI Elements** | **Problem Description** | **1point** | **2points** | **3points** | **4points** |
| --- | --- | --- | --- | --- | --- |
| **Fitness equipment (e.g., joints, mounting points)** | **Do you find yourself repeatedly examining the structural components of a piece of equipment because you’re worried it might be unstable or pose a safety hazard?** | Never **□** | Occasionally **□** | Frequently **□** | Always **□** |
| **Equipment Usage Instructions Sign** | **Do you need to read (or look back at) the instructions or illustrations repeatedly to understand how to use it?** | Never **□** | Occasionally **□** | Frequently **□** | Always **□** |
| **Resting Seats** | **Is the seat's color too similar to its surroundings, making it hard to spot at first glance?** | Never **□** | Occasionally **□** | Frequently **□** | Always **□** |
| **Floor Surface (Under and Around the Equipment)** | **Are you worried that the floor might be slippery or uneven, causing you to keep looking down at your feet while using the equipment?** | Never **□** | Occasionally **□** | Frequently **□** | Always **□** |
| **Overall Environment (Color and Lighting)** | **Do the bright colors or strong light contrasts on the equipment cause your eyes to feel strained or tired?** | Never **□** | Occasionally **□** | Frequently **□** | Always **□** |

**（3）Second Interview (Please answer the following questions based on your eye-tracking data)**

**1.** Follow-up question regarding the behavior of staring: You were looking at a certain piece of equipment repeatedly just now—was it because it looked interesting, safe, or because you didn’t understand how to use it?

_____________________________________________________________________

_____________________________________________________________________

_____________________________________________________________________

2. Follow-up on safety concerns: Are you repeatedly looking at the connection point because you’re worried it might be unstable? Has this affected your willingness to use it?

_____________________________________________________________________

_____________________________________________________________________

_____________________________________________________________________

3. Identify the cause of eye strain: If you’re experiencing eye strain, is it due to excessive color contrast, glare, or an overload of information?

_____________________________________________________________________

_____________________________________________________________________

**2**. **Rest and Communication Area**

**（1）Subjective Satisfaction Questionnaire (**Scoring Guide: 1 = Very Dissatisfied, 2 = Somewhat Dissatisfied, 3 = Neutral, 4 = Somewhat Satisfied, 5 = Very Satisfied**)**

| **No.** | **Review Content** | **1point** | **2points** | **3points** | **4points** | **5points** |
| --- | --- | --- | --- | --- | --- | --- |
| **1** | **Seat comfort (backrest angle, armrest height, seat firmness, and width)** | **□** | **□** | **□** | **□** | **□** |
| **2** | **Practicality of Sunshade and Rain Shelter Facilities (Coverage Area, Structural Sturdiness, Durable Materials)** | **□** | **□** | **□** | **□** | **□** |
| **3** | **Does the spatial layout facilitate conversation among acquaintances without exposing them too much to the gaze of strangers?** | **□** | **□** | **□** | **□** | **□** |
| **4** | **Does the quality of the surrounding landscaping create a relaxing atmosphere, and does it feature pleasing seasonal changes?** | **□** | **□** | **□** | **□** | **□** |
| **5** | **Does the seat color stand out clearly against the surroundings, making it easy to locate and identify?** | **□** | **□** | **□** | **□** | **□** |
| **6** | **Do the sunshades and rain shelters cover some of the equipment, making it easier to use in different weather conditions?** | **□** | **□** | **□** | **□** | **□** |
| **7** | **A balance between visual privacy and openness, offering a sense of support or enclosure without feeling confined** | **□** | **□** | **□** | **□** | **□** |
| **8** | **Amenities: Are there any places nearby to set down water cups or canes, or small tables?** | **□** | **□** | **□** | **□** | **□** |
| **9** | **Accessibility: Is the path from the main route to the seating area level and free of elevation changes?** | **□** | **□** | **□** | **□** | **□** |
| **10** | **Condition of the facility: Are the seats clean and free of damage? Is the surrounding area tidy?** | **□** | **□** | **□** | **□** | **□** |

**（2）Eye Movement Correlation Questionnaire (Each question has equidistant quantitative response options, such as “Never,” “Occasionally,” “Often,” and “Always,” corresponding to scores of 1–4)**

| **AOI Elements** | **Problem Description** | **1point** | **2points** | **3points** | **4points** |
| --- | --- | --- | --- | --- | --- |
| **Reclining Chair** | **Can you quickly find a chair to sit on in your surroundings?** | Never **□** | Occasionally **□** | Frequently **□** | Always **□** |
| **Edges of awnings/pavilion canopies** | **Does the edge of the canopy significantly obstruct your view of the surroundings when you are seated?** | Never **□** | Occasionally **□** | Frequently **□** | Always **□** |
| **Flower Beds/Landscaping (Reflective Plants or Ornaments)** | **Are there certain plants or decorative items whose reflections are so glaring that you don't want to look at them for too long?** | Never **□** | Occasionally **□** | Frequently **□** | Always **□** |
| **The junction between the seat and the floor** | **When you sit down or stand up, do you make a point of checking whether the floor around the chair legs is level?** | Never **□** | Occasionally **□** | Frequently **□** | Always **□** |

**（3）Second Interview (Please answer the following questions based on your eye-tracking data)**

**1.** Ask follow-up questions about the comparison: When you keep comparing seats, are you considering the location, comfort, or your distance from others?

_____________________________________________________________________

_____________________________________________________________________

_____________________________________________________________________

**2.** Follow-up question on sustained gaze: Do you keep looking at a certain spot because it’s beautiful, interesting, or because it makes you feel safe?

_____________________________________________________________________

_____________________________________________________________________

_____________________________________________________________________

**3.** Follow-up question regarding visual discomfort: If you feel that the sunshade is blocking your view, would that cause you to choose not to sit here?

_____________________________________________________________________

_____________________________________________________________________

_____________________________________________________________________

**3. Campus Walkway Area**

**（1）Subjective Satisfaction Questionnaire (**Scoring Guide: 1 = Very Dissatisfied, 2 = Somewhat Dissatisfied, 3 = Neutral, 4 = Somewhat Satisfied, 5 = Very Satisfied**)**

| **No.** | **Review Content** | **1point** | **2points** | **3points** | **4points** | **5points** |
| --- | --- | --- | --- | --- | --- | --- |
| **1** | **Skid resistance and overall smoothness of pavement materials** | **□** | **□** | **□** | **□** | **□** |
| **2** | **Are road boundaries (such as curbs and the edges of green belts) clear, continuous, and easily distinguishable?** | **□** | **□** | **□** | **□** | **□** |
| **3** | **Are the slopes and steps designed to be gentle? Are there handrails? Are the steps of a moderate height?** | **□** | **□** | **□** | **□** | **□** |
| **4** | **Are there safe and clear separations between the walkway and the surrounding areas (roadways, activity areas)?** | **□** | **□** | **□** | **□** | **□** |
| **5** | **How clear is the line of sight at the turn? Has vegetation or other structures obstructing the view been removed in advance?** | **□** | **□** | **□** | **□** | **□** |
| **6** | **Are rest stops properly located? Are the distances between them reasonable? Are there seats available for resting?** | **□** | **□** | **□** | **□** | **□** |
| **7** | **Is the scenery along the route interesting? Are there diverse landscaping elements or small features to avoid monotony?** | **□** | **□** | **□** | **□** | **□** |
| **8** | **Safety considerations: Is the trail separate and quiet, free from interference from vehicles or fast cyclists?** | **□** | **□** | **□** | **□** | **□** |
| **9** | **Is the width sufficient to allow two people to walk side by side or pass each other, or for someone using a wheelchair or walker to pass through?** | **□** | **□** | **□** | **□** | **□** |
| **10** | **Visual illusions on the ground: Could differences in pattern or material color lead to misjudgments regarding height or levelness?** | **□** | **□** | **□** | **□** | **□** |

**（2）Eye Movement Correlation Questionnaire (Each question has equidistant quantitative response options, such as “Never,” “Occasionally,” “Often,” and “Always,” corresponding to scores of 1–4)**

| **AOI Elements** | **Problem Description** | **1point** | **2points** | **3points** | **4points** |
| --- | --- | --- | --- | --- | --- |
| **Sidewalk** | **Do you find yourself frequently looking down because you're worried the road might be slippery, cracked, or uneven?** | Never **□** | Occasionally **□** | Frequently **□** | Always **□** |
| **Curb/Greenbelt Boundary** | **Is the edge of the sidewalk (where it meets the lawn or the roadway) clearly defined? Do you have to turn your head to see it?** | Never **□** | Occasionally **□** | Frequently **□** | Always **□** |
| **Visibility around corners (e.g., bushes, corners of walls)** | **At a turn in the road, is there something blocking your view that makes you stop and take a closer look before continuing on?** | Never **□** | Occasionally **□** | Frequently **□** | Always **□** |

**（3）Second Interview (Please answer the following questions based on your eye-tracking data)**

**1.** Follow-up Question on Road Surface Awareness: Do you keep looking at the road surface because you’re worried about slipping or tripping, or because of visual misperceptions?

_____________________________________________________________________

_____________________________________________________________________

_____________________________________________________________________

**2.** Ask follow-up questions about turning behavior: Is the driver looking ahead at the turn because their view is obstructed, or because the path is unclear?

_____________________________________________________________________

_____________________________________________________________________

_____________________________________________________________________

**3.** Ask why they feel nervous: If they feel nervous, is it because of heavy foot traffic, traffic disruptions, or poor lighting?

_____________________________________________________________________

_____________________________________________________________________

_____________________________________________________________________

**4.** **Multi-functional Plaza Area**

**（1）Subjective Satisfaction Questionnaire (**Scoring Guide: 1 = Very Dissatisfied, 2 = Somewhat Dissatisfied, 3 = Neutral, 4 = Somewhat Satisfied, 5 = Very Satisfied**)**

| **No.** | **Review Content** | **1point** | **2points** | **3points** | **4points** | **5points** |
| --- | --- | --- | --- | --- | --- | --- |
| **1** | **Does the sense of spaciousness make one feel free and unconfined?** | **□** | **□** | **□** | **□** | **□** |
| **2** | **Convenience for participating in group activities (square dancing, tai chi, board and card games)** | **□** | **□** | **□** | **□** | **□** |
| **3** | **Does the seating arrangement encourage face-to-face interaction among multiple people and create a small-group atmosphere?** | **□** | **□** | **□** | **□** | **□** |
| **4** | **The cleanliness and aesthetic appeal of the overall environment** | **□** | **□** | **□** | **□** | **□** |
| **5** | **The slip resistance of the flooring material, and whether color variations or glare cause visual misperceptions** | **□** | **□** | **□** | **□** | **□** |
| **6** | **Are the activity areas and passageways clearly demarcated so that they do not interfere with one another?** | **□** | **□** | **□** | **□** | **□** |
| **7** | **Does the design of the plaza help alleviate noise buildup to some extent?** | **□** | **□** | **□** | **□** | **□** |
| **8** | **Visual focal points: Are there any attractive views or features that serve as visual anchors?** | **□** | **□** | **□** | **□** | **□** |
| **9** | **Adaptability: Whether it can meet various needs—such as sitting still, observing, and being active—at different times** | **□** | **□** | **□** | **□** | **□** |
| **10** | **Accessible design: Is it easy to enter the plaza from all directions? Are there accessible pathways?** | **□** | **□** | **□** | **□** | **□** |
| **11** | **Sense of security: Is the plaza within the coverage area of the community’s security surveillance system or under natural surveillance?** | **□** | **□** | **□** | **□** | **□** |

**（2）Eye Movement Correlation Questionnaire (Each question has equidistant quantitative response options, such as “Never,” “Occasionally,” “Often,” and “Always,” corresponding to scores of 1–4)**

| **AOI Elements** | **Problem Description** | **1point** | **2points** | **3points** | **4points** |
| --- | --- | --- | --- | --- | --- |
| **The boundary between the event area and the pedestrian area in the plaza** | **Can you tell at a glance which areas are for dancing and which are for walking through?** | Never **□** | Occasionally **□** | Frequently **□** | Always **□** |
| **Seats arranged in groups** | **Does the way the seats are arranged make you feel like it’s easy to join in on other people’s conversations, or would you rather sit quietly by yourself?** | Never **□** | Occasionally **□** | Frequently **□** | Always **□** |
| **Changes in the ground patterns or materials in the center of the plaza** | **Do patterns on the ground or differently colored floor tiles ever make you think there are steps or potholes there?** | Never **□** | Occasionally **□** | Frequently **□** | Always **□** |

**（3）Second Interview (Please answer the following questions based on your eye-tracking data)**

**1**. Ask about the first thing you noticed: Did you look there first because it stood out or caught your interest, or because you had a specific goal in mind?

_____________________________________________________________________

_____________________________________________________________________

_____________________________________________________________________

**2.** Follow-up question on sustained attention: Do you keep your attention focused on a particular activity because you want to participate or observe, or because it makes you feel safe?

_____________________________________________________________________

_____________________________________________________________________

_____________________________________________________________________

**3**. Follow-up on visual clutter: Does the visual clutter make you feel confused or anxious? Does it affect your willingness to stay here?

_____________________________________________________________________

_____________________________________________________________________

_____________________________________________________________________

**5. Greened Landscape Area**

（1） **Subjective Satisfaction Questionnaire (**Scoring Guide: 1 = Very Dissatisfied, 2 = Somewhat Dissatisfied, 3 = Neutral, 4 = Somewhat Satisfied, 5 = Very Satisfied**)**

| **No.** | **Review Content** | **1point** | **2points** | **3points** | **4points** | **5points** |
| --- | --- | --- | --- | --- | --- | --- |
| **1** | **The ornamental value of plants and the visual freshness brought by seasonal changes** | **□** | **□** | **□** | **□** | **□** |
| **2** | **Are there any plant arrangements designed to be easily observed, touched, or smelled up close?** | **□** | **□** | **□** | **□** | **□** |
| **3** | **Does the integration of landscaping and recreational facilities appear natural and harmonious, and does it provide shade?** | **□** | **□** | **□** | **□** | **□** |
| **4** | **Does the combination of plant colors create a sense of harmony, comfort, and tranquility?** | **□** | **□** | **□** | **□** | **□** |
| **5** | **Are there any materials that cause glaring reflections (such as water features, metal accents, or glass)?** | **□** | **□** | **□** | **□** | **□** |
| **6** | **Do the plants obstruct pedestrian paths, important signs, or surveillance views?** | **□** | **□** | **□** | **□** | **□** |
| **7** | **Are there any iconic plants or landmarks that make it easy to recognize the changing seasons?** | **□** | **□** | **□** | **□** | **□** |
| **8** | **Is the sense of depth in the landscape (foreground, middle ground, and background) clear and rich?** | **□** | **□** | **□** | **□** | **□** |
| **9** | **Maintenance condition: Are the plants neatly trimmed, free of dead branches and leaves, and are the landscape features in good condition?** | **□** | **□** | **□** | **□** | **□** |
| **10** | **Is it eco-friendly? Does it attract birds, butterflies, and other creatures, adding vitality and interest?** | **□** | **□** | **□** | **□** | **□** |
| **11** | **The appeal of the path: Are the trails winding through the landscape both interesting and safe?** | **□** | **□** | **□** | **□** | **□** |
| **12** | **Does the sense of tranquility in the overall environment help you relax and relieve stress?** | **□** | **□** | **□** | **□** | **□** |

**（2）Eye Movement Correlation Questionnaire (Each question has equidistant quantitative response options, such as “Never,” “Occasionally,” “Often,” and “Always,” corresponding to scores of 1–4)）**

| **AOI Elements** | **Problem Description** | **1point** | **2points** | **3points** | **4points** |
| --- | --- | --- | --- | --- | --- |
| **Path signs obscured by tree branches and leaves** | **Are there any directional signs that are obscured by branches and leaves, making them hard to find?** | Never **□** | Occasionally **□** | Frequently **□** | Always **□** |
| **Shrubs or the edges of lawns that spread onto walking paths** | **Do you often have to watch your step when walking to avoid tripping over overhanging branches?** | Never **□** | Occasionally **□** | Frequently **□** | Always **□** |
| **Reflective features such as water features and metal decorations** | **Do highly reflective decorative elements in a landscape make your eyes feel uncomfortable, causing you to look away?** | Never **□** | Occasionally **□** | Frequently **□** | Always **□** |
| **Combining Plants of Different Colors** | **Does the overall color scheme of the flowers, plants, and trees here make you feel relaxed and happy?** | Never **□** | Occasionally **□** | Frequently **□** | Always **□** |

**（3）Second Interview (Please answer the following questions based on your eye-tracking data)**

1. Follow-up question on what attracts you: Does this plant attract you because of its vibrant colors and beautiful shape, or because it evokes a certain memory for you?

_____________________________________________________________________

_____________________________________________________________________

_____________________________________________________________________

2. Follow-up question on obstructions: Do plants blocking signs ever confuse or unsettle you? How do you handle such situations?

_____________________________________________________________________

_____________________________________________________________________

_____________________________________________________________________

3. Ask yourself about visual fatigue: If you feel fatigued, is it because the colors are too intense, the depth of field is confusing, or because of information overload?

_____________________________________________________________________

_____________________________________________________________________

_____________________________________________________________________

**6. Public Facility Node Area**

**（1）Subjective Satisfaction Questionnaire (**Scoring Guide: 1 = Very Dissatisfied, 2 = Somewhat Dissatisfied, 3 = Neutral, 4 = Somewhat Satisfied, 5 = Very Satisfied**)**

| **No.** | **Review Content** | **1point** | **2points** | **3points** | **4points** | **5points** |
| --- | --- | --- | --- | --- | --- | --- |
| **1** | **Sign Visibility (Location, Size, Color) and Information Comprehensibility** | **□** | **□** | **□** | **□** | **□** |
| **2** | **The cleanliness and ease of locating facilities such as trash cans and water fountains** | **□** | **□** | **□** | **□** | **□** |
| **3** | **Does the space feel open, or are there any visual obstructions or elements that create a sense of confinement?** | **□** | **□** | **□** | **□** | **□** |
| **4** | **Convenience and comfort as a place for brief stops and chance encounters** | **□** | **□** | **□** | **□** | **□** |
| **5** | **Is the layout of the various facilities orderly, avoiding visual clutter and confusion?** | **□** | **□** | **□** | **□** | **□** |
| **6** | **Are service information signs (such as directions and distances to public restrooms) clear and effective?** | **□** | **□** | **□** | **□** | **□** |
| **7** | **Protection from sun and rain: Are there canopies or tree shade at key locations where people can take brief shelter?** | **□** | **□** | **□** | **□** | **□** |
| **8** | **Handling of elevation differences: Are all areas level and free of hazards, making it easy to stand?** | **□** | **□** | **□** | **□** | **□** |
| **9** | **In addition to visual signage, has the multimodal communication of information taken tactile elements (such as tactile paving) into account?** | **□** | **□** | **□** | **□** | **□** |
| **10** | **The clarity of a decision point—does it allow people to quickly determine the next steps?** | **□** | **□** | **□** | **□** | **□** |

**（2）Eye Movement Correlation Questionnaire (Each question has equidistant quantitative response options, such as “Never,” “Occasionally,” “Often,” and “Always,” corresponding to scores of 1–4)**

| **AOI Elements** | **Problem Description** | **1point** | **2points** | **3points** | **4points** |
| --- | --- | --- | --- | --- | --- |
| **General Information Signs (Maps, Directional Signs)** | **Can you spot this sign right away and quickly understand what it says?** | Never **□** | Occasionally **□** | Frequently **□** | Always **□** |
| **Trash Can** | **Do the color and placement of the trash cans make them easy to spot?** | Never **□** | Occasionally **□** | Frequently **□** | Always **□** |
| **Minor elevation differences on the ground (such as manhole covers and pavement joints)** | **Do you pay special attention to whether there are any sudden small steps or uneven spots on the ground beneath your feet?** | Never **□** | Occasionally **□** | Frequently **□** | Always **□** |
| **Layout of all facilities in the entire node area** | **Do the various signs and facilities at this intersection/entrance look cluttered when placed together? Do they make you feel overwhelmed by too much information?** | Never **□** | Occasionally **□** | Frequently **□** | Always **□** |

**（3）Second Interview (Please answer the following questions based on your eye-tracking data)**

1. Follow-up Question on Sign Recognition: Is the sign difficult to spot because of its location, size, or color, or because it blends in too well with its surroundings?

_____________________________________________________________________

_____________________________________________________________________

2. Ask follow-up questions to identify comprehension difficulties: If you need to reread the text repeatedly, is it because the font is too small, the language is too complex, or the information is disorganized?

_____________________________________________________________________

_____________________________________________________________________

3. Ask about visual stress: Does visual stress make you want to leave the area as soon as possible? Does it affect your willingness to use the facilities?

_____________________________________________________________________

_____________________________________________________________________

**III. Overall Experience and Recommendations**

**1. Overall, how would you rate the visual environment (clarity, comfort, and aesthetics) of the outdoor spaces on campus?**

**□** Very comfortable **□** Fairly comfortable **□** Average **□** Not very comfortable **□** Very chaotic/uncomfortable

**2. Based on today’s experience, which of the above types of areas do you think needs the most improvement in terms of visual design and user-centered considerations?**

**□** sports and fitness area **□** rest and communication area **□** campus walkway area **□** multi-functional plaza area **□** greened landscape area **□** public facility node area

**3. What specific suggestions do you have for making the venue more youth-friendly in terms of its visual appeal?**

**① In the space you just experienced, what element or design most drew you to stop and look? Why?**

**_____________________________________________________________________**

**_____________________________________________________________________**

**② Based on your observations and usage habits, what is the most pressing visual or spatial design issue that needs to be addressed at this site?**

**_____________________________________________________________________**

**_____________________________________________________________________**

**③ What other suggestions or ideas do you have for creating outdoor campus spaces that better meet students’ behavioral and psychological needs?**

**_____________________________________________________________________**

**_____________________________________________________________________**
