# Supplementary material for: A study on the visual behavior and cognitive preferences of female college students in outdoor campus spaces based on eye-tracking
Source: Front Psychol. 2026 Sep 3;17:1896634. doi: 10.3389/fpsyg.2026.1896634 (PMC13581501; doi:10.3389/fpsyg.2026.1896634)
Supplement: Supplementary file 2 [file Table_1.DOCX]

Supplementary Material

# 1 Supplementary Data

# Upload the raw data separately: name it as eye movement data

# 2 Supplementary Figures and Tables

**Table 5．**Comparison of Eye-Tracking Metrics Across 6 Types of Venues

| Site Type | Total Fixation Duration (S) Mean ± Standard Deviation | Average Fixation Duration (S) Mean ± standard deviation | First Fixation Duration (S) Mean ± Standard Deviation | Fixation Count (times) Mean ± Standard Deviation |
| --- | --- | --- | --- | --- |
| Sports and Fitness Area | 72.36±28.54 | 0.21±0.08 | 0.16±0.07 | 344.59±136.27 |
| Rest and Communication Area | 56.82±24.31 | 0.15±0.06 | 0.12±0.05 | 278.47±112.63 |
| Campus Walkway Area | 228.65±89.72 | 0.38±0.15 | 0.28±0.12 | 601.76±235.49 |
| Multi-functional Plaza Area | 89.74±36.85 | 0.18±0.07 | 0.14±0.06 | 402.35±158.91 |
| Greened Landscape Area | 215.38±85.64 | 0.36±0.14 | 0.26±0.11 | 578.24±224.78 |
| Public Facility Node Area | 98.41±42.16 | 0.19±0.08 | 0.15±0.07 | 426.59±173.84 |

**Table 8** Correlation Analysis Between Eye-Tracking Metrics and Overall Venue Satisfaction (r-value)

| **Visual Objectives** | Total Fixation Duration**（TFD）** | Average Fixation Duration**（AFD）** | First Fixation Duration**（FFD）** | Fixation Count**（FC）** |
| --- | --- | --- | --- | --- |
| trees | 0.683** | 0.597** | 0.482** | 0.651** |
| grassland | 0.328* | 0.294* | 0.276* | 0.315* |
| architecture | 0.187 | 0.153 | 0.129 | 0.174 |
| road surface | -0.425** | -0.389** | -0.357** | -0.408** |
| sign | -0.396** | -0.348** | -0.312* | -0.377** |
| guardrails | -0.269* | -0.241 | -0.228 | -0.256 |
| seats | 0.214 | 0.198 | 0.183 | 0.207 |
| pedestrians | -0.172 | -0.156 | -0.143 | -0.168 |
| streetlights | 0.087 | 0.079 | 0.068 | 0.083 |
| pond | 0.092 | 0.085 | 0.076 | 0.089 |
| stone tables | 0.064 | 0.058 | 0.051 | 0.061 |
